# Vehicle choice modeling for emerging zero-emission light-duty vehicle markets in California

**DOI:** 10.1016/j.heliyon.2024.e32823

**Published:** 2024-06-16

**Authors:** Andrew F. Burke, Jingyuan Zhao, Marshall R. Miller, Lewis M. Fulton

**Affiliations:** Institute of Transportation Studies, University of California Davis, Davis, CA, USA

**Keywords:** Battery electric vehicle, Fuel cell vehicle, Light-duty vehicle, Market, Consumer preference, Policy

## Abstract

To predict the market dynamics of various zero-emission vehicle (ZEV) technologies, this study introduces a dynamic discrete vehicle choice model (VCM) that investigates the probabilities associated with 14 decision factors, applying these to the purchase of ZEVs from 2020 to 2040. Market share and penetration results are presented under eight scenarios, that vary by vehicle costs infrastructure development and incentive strategies. The findings suggest that in the early years, incentives alone may not generate significant market penetration of ZEVs before the infrastructure meets the basic convenience for daily use, especially for fuel cell vehicles (FCVs). However, in later years, incentives play a more important role in the market penetration of ZEVs under well-defined infrastructure networks. By 2040, battery electric vehicles (BEVs) are projected to dominate the market in California. Plug-in hybrid electric vehicles (PHEVs) and FCVs may experience a decline in market share due to improved charging convenience, which benefits the market penetration of BEVs. However, fuel cell plug-in hybrid electric vehicles (FC-PHEVs) could still be beneficial if accessible models are available, considering the limited availability of hydrogen refueling stations. The goal set by the California Air Resources Board (CARB) is achievable, but it requires a sustained combination of measures; no single effort can achieve it. These measures include technological improvements to reduce the cost of ZEVs, a wider range of models available for consumers to choose from based on their desired performance, the establishment of infrastructure (battery chargers and hydrogen dispensers), and attractive incentives aimed at promoting ZEV adoption. The proposed methodology can be adapted for other regions in the United States and globally by carefully examining the inputs for each decision factor at the desired scale.

## Highlights

• A dynamic discrete choice model is developed for purchase probability analysis.
• Discrete choice model is conducted for cars, SUVs and light-duty pickups.
• The model examines 14 ZEV purchase decision attributes spanning from 2020 to 2040.
• Eight scenarios are designed to investigate the market penetration of BEVs and FCVs.
• Results emphasize the importance of combined measures and policies for ZEV market.

## 1 Introduction

The current adoption of zero-emission vehicles (ZEVs) is quite limited and is currently dominated by battery-electric vehicles (BEV) using lithium-ion batteries. The widespread adoption of ZEVs in the global automotive market faces a long journey ahead with many challenges to be overcome [[1], [2], [3], [4]]. California has established regulations stating that by 2035 for light-duty vehicles (LDVs) [5,6], all vehicles sold must be either BEV or fuel cell vehicles (FCV). Consequently, engine-powered vehicles will no longer be available for purchase. To achieve this goal, significant efforts have been made over the past several years. California leads the nation in EV adoption, with a substantial portion of new light-duty ZEVs being sold in the state. Access to information and understanding real market penetration are crucial for comprehending the California market. For example, in 2023, California accounted for 34 % of all new light-duty ZEV sales in the U.S. Additionally, about 25 % of all new cars sold in California that year, which equals 446,961 vehicles, were zero emission [7]. According to the California Energy Commission (CEC) data as of January 2024 [8], there are 105,012 EV chargers available, of which 41.28 % are public and 58.72 % are shared private chargers. There are also 68 light-duty hydrogen stations, with plans for 34 more.

**Fig. 1:**
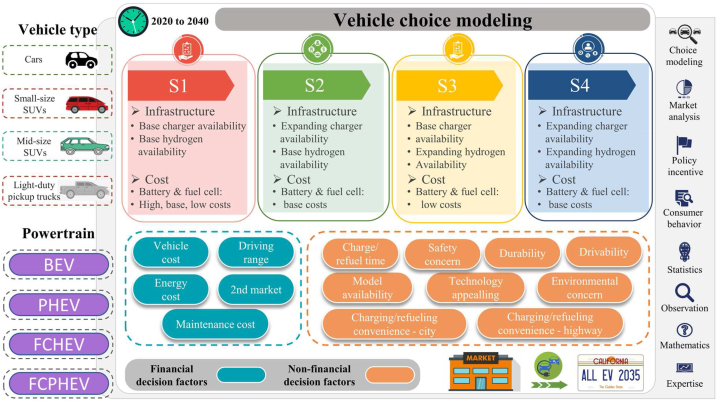
The framework of dynamic discrete choice modeling for purchase probability analysis.

Questions to be addressed in this paper are what will be the market shares of the ZEVs between now and 2040 and is it likely that the mass market can be met by all types of ZEVs. The dynamic discrete choice (DDC)-based model (Fig. 1) was developed to assess how buyers decide whether they want to purchase a ZEV rather than the engine-powered vehicles they would normally be buying. Totally fourteen decision factors are investigated for the modeling of LDV adoption. All buyers will expect that the ZEVs will meet their needs at least as well as the engine-powered vehicles they are currently using. The vehicle choice model calculate the probability that the buyers will decide to purchase one of the ZEV technologies based on the sub-probabilities associated with fourteen decision factors. The DDC model presented in this paper has been applied to California markets because the State has been a world leader in promoting sales of ZEVs. The method can be applied to other regions within the United States and to countries worldwide by modifying the inputs for each decision factor at the desired scale. Vehicle choice modeling is concerned with projecting/predicting what vehicles new car buyers will purchase when given choices that include ZEVs technologies and alternative fuels. The present project involved battery electric and hydrogen FCVs in competition with gasoline and diesel engine vehicles.

Vehicle choice models have been under development widely in connection with assessing the marketability of vehicles using alternative fuels to reduce emissions and the use of petroleum. For example, Oak Ridge National Lab conducted an early study that described a multinomial logit-type model for analyzing the alternative fuels market [9]. Following this study, subsequent research has been dedicated to vehicle choice modeling [[10], [11], [12]], and their latest model and software, MA3T (Market Acceptance of Advanced Technologies) [13], are considered among the most significant studies available. That model, along with other multinomial logit programs, makes it difficult to identify the inputs used in their analysis due to factors such as the large number of potential input variables and the lack of transparency in model documentation. One of the objectives of this study is to develop a simpler approach where the inputs in the model are easier to follow.

The 14 decision factors used in the DDC model are not much different than those that have been used in previous vehicle choice models [[14], [15], [16], [17], [18]]. Some of the factors are calculated from detailed calculations of vehicle cost and performance at specified years in the future out to 2040. Other factors are more subjective and are estimated based on the literature and the experience of the model user. Most of the factors relate the characteristics and market situations of ZEV and ICEV and apply to LDVs. This paper discusses in detail the model approach, inputs, and results obtained using the model to project the ZEV markets in California for 2020–2040. Market share results are given for multiple types of LDVs.

The structure of this paper consists of five principal sections. Initially, we review past approaches to vehicle choice modeling. This offers a foundation for understanding historical methods in the field and uncovers certain limitations of past models. Next, we introduce the new approach to vehicle choice modeling, designed to overcome the deficiencies of previous methods and to project vehicular market dynamics more easily for various types of ZEVs. Following this, we present results obtained using the DDC-based approach for purchase probability analysis (PPA) model under different scenarios, encompassing varying policy environments and market conditions. The results provide an in-depth understanding of how the model functions in practice and the usefulness of the model to analysis different market conditions. In the fifth section, we compare market share projections between the UC Davis PPA model and the models developed at the Department of Energy National Laboratories and with milestones/mandates of the California Air Resources Board (CARB). Through these comparisons, we assess the performance of the PPA model and suggest avenues for its improvement in the future. In short, this study offers several contributions:

(i) We introduce a DDC model specifically designed for PPA. This model is effective at predicting consumer decisions related to vehicle purchases, covering from cars and SUVs to light-duty pickup trucks. It takes advantage of recent developments in the ZEV market in California. Beyond its new concepts, this research details potential market distributions across eight hypothetical scenarios, ensuring they align with the CARB's goals for ZEVs.
(ii) The research examines 14 important attributes that influence decisions regarding ZEV purchases. Such an examination helps us understand the factors driving these choices from 2020 to 2040. The study provides insights into the market penetration of battery-electric plug-in vehicles by the year 2030. It also highlights the potential introduction and acceptance of hydrogen FCVs in the market landscape.
(iii) Although this study is based in the California context, it demonstrates the flexibility of the PPA methodology. With necessary adjustments, this technique could be relevant and suitable for different infrastructure setups and policies in various regions.

## 2 Past approaches to vehicle choice modeling

The DDC model [9,[12], [13], [14], [15], [16], [17], [18]] is one of the most prominent approaches in economic modeling to represent consumer choice decisions. DDC models typically assume that aggregate agent expectations are rational expectations, which is a widely accepted assumption in economic modeling. Such choice models provide insights into the factors that drive individual decision-making and can be used to predict choice behavior under different scenarios or policy changes. They allow researchers to quantify the influence of different attributes, evaluate the relative importance of alternatives, and analyze market shares or demand for different options. Most of the past approaches to vehicle choice modeling were based on the nested multinomial logit method approach. The nested multinomial logit model [9] is a specific type of discrete choice model that incorporates a nested structure among the alternatives. It extends the standard multinomial logit (MNL) model by allowing for correlation and heterogeneity within groups or nests of alternatives. Comprehensive review of these methods applied to LDVs is given in Refs. [[19], [20], [21], [22]].

In the multinomial logit method, the generalized lifetime costs of various types of vehicles using a wide range of powertrains and energy storage technologies are calculated. The resultant cost differences/ratios between the vehicle options are used to project the sales fraction of each of the vehicles as the performance and costs of the different technologies matures in future years. The generalized costs include both cost components such as initial vehicle cost, maintenance cost, and fuel cost that are normally expressed in monetary ($) terms, but also the estimated monetary values to consumers of subjective purchase factors for which monetary value is not customarily assigned. These subjective factors include range anxiety, limited refilling infrastructure, inconvenient refilling time, and limited availability of vehicle models using the new technologies. The value to the consumer of these latter decision factors is necessarily subjective for all vehicle choice approaches and dependent on the judgement of the model user. In most existing models of vehicle choice [14,21], the subjective inputs are difficult to determine from the papers describing them and rather arbitrary in magnitude. In this paper, all the inputs for the PPA approach are clearly identified and the values of all the factors for typical calculations listed. The inputs for different States and countries are expected to be different than those shown for California.

Diverse factors influence the market dynamics and customer predilections towards ZEVs. They span across multiple factors, including the availability of ZEV models, fuel/energy alternatives, market-specific scenarios, battery charging networks, and considerations related to hydrogen production and distribution. Consumer attitudes towards ZEVs are shaped by an array of elements such as age, educational level, geographic location, marital status, and income [23,24]. Crucial factors influencing potential ZEV buyers encompass affordability, performance, driving range, refueling time, and environmental concerns [[25], [26], [27]]. The principal obstacles impeding broader ZEV acceptance are high initial costs and subpar range and difficulty in refueling compared to gasoline ICEVs. To date, a comprehensive set of incentives targeting vehicle buyers seems to be crucial to foster widespread acceptance of ZEVs [[28], [29], [30]]. Government policies to support ZEV manufacture and purchase have included sales mandates for manufacturers and financial incentives for vehicle buyers. Adoption of BEVs have been most successful in California [28], Norway [29], Sweden [31], and China [32]. In all those places, government policies played the key role in driving BEV sales. It is apparent that economic factors and vehicle user convenience are critical in influencing LD vehicle purchasing decisions in all countries. In California, a tripartite strategy incorporating stringent ZEV regulations, consumer incentives, and public-private partnerships to build battery charging and hydrogen stations has been employed with limited, but increasing success, to promote ZEV sales. Much has been learned in recent years concerning decision factors for sales of LD ZEVs.

Specifically, specialized models have been designed worldwide, based on the discrete choice model. For instance, one study investigates the economic implications of simultaneously enforcing the Corporate Average Fuel Economy (CAFE) standards, greenhouse gas (GHG) emissions regulations, and ZEV requirements in the U.S. Using the "Cost Optimization Modeling for Efficiency Technologies" (COMET) model [33], it assesses how manufacturers can meet these diverse regulations. Key findings indicate that by 2025, compliance costs per vehicle will be around $1600 for CAFE/GHG and $2000 for both CAFE/GHG and ZEV. Notably, the combined cost for dual compliance is less than the sum of individual compliances, suggesting economic benefits from integrated strategies. This study highlights the complex interplay and economic impact of automotive regulations, advocating for holistic compliance approaches. Focusing also on the U.S. market, the vehicle electrification group at Carnegie Mellon University [34] has conducted a broad range of studies using choice models, covering various research areas. These include: Technology, Life-cycle, Consumer Behavior, Public Policy: The group produces policy-relevant technical findings and policy analysis to inform decision-making in this field. One of its recent studies implies that technological advancements in BEVs are playing a crucial role in shaping consumer preferences and market trends [35]. It highlights the potential for a significant market shift towards electric vehicles, driven primarily by improvements in BEV technology. This study underscores the importance of continued technological innovation in BEVs and suggests a nearing point where BEVs could dominate new vehicle sales, propelled by consumer preferences and technological advancements.

Similarly, another study focuses on Canada's LDV sector, using the Automaker-consumer Model (AUM) to analyze various ZEV mandate designs [36], targeting 30 % ZEV sales by 2030. It examines the impact on ZEV adoption, GHG emissions, consumer surplus, automaker profits, and policy cost-effectiveness. The study finds that a higher non-compliance penalty (CDN$ 10,000 per credit) is crucial for meeting ZEV targets. A “One credit per ZEV” scheme is more effective than a “California-style” multi-credit system, and credit banking helps soften profit impacts and encourages early compliance. The most cost-effective approach combines a $10,000 penalty with a single-credit system and credit banking. This research highlights the importance of policy structure in the automotive sector, revealing the trade-offs in environmental policy design. Further discussion of these models is available in several in-depth analysis review papers [37,38], which provide detailed insights into the subject.

## 3 Dynamic discrete choice-based vehicle choice modeling

### 3.1 Basis of the dynamic discrete choice model

The heterogeneous preferences of consumers or fleets when making decisions about which cars or pickup trucks to buy and drive will be a critical determinant of the model. Here, we develop some representations of consumer preferences in the energy-economy space, specifically focusing on both financial and non-financial preferences of individuals and fleets. The PPA approach includes the specific decision factors shown in Table 1. These factors are discussed further in **Appendix A**.

**Table 1:** Decision factors for the purchase of vehicles using various technology options.

No.	Attribute
1	Vehicle cost
2	All-electric or hydrogen driving range (mi)
3	Number of models available to purchase
4	Inconvenience to charge or refuel ZEVs in the city compared to ICEVs
5	Inconvenience to charge or refuel ZEVs on the highway compared to ICEVs
6	Battery charging or hydrogen refueling time (minutes)
7	Availability of a second market for ZEVs compared to ICEVs
8	Maintenance cost ($/mi)
9	Energy operating cost ($/mi)
10	Environmental concern compared to ICEVs
11	Safety concern compared to ICEVs
12	Drivability of ZEVs compared to ICEVs
13	Reliability/durability of ZEVs compared to ICEVs
14	Excitement with the ZEVs technologies compared to ICEVs

### 3.2 The formulation of the purchase probability analysis approach

This section of the paper presents in detail the analytical framework of the PPA model and how the market share results are calculated in EXCEL spreadsheets. The probability ($P_{r}$) that a particular decision factor will favorably or unfavorably influence the vehicle purchase is assumed to be of the form:

(1) $$P_{r}=e^{a\left(1-1/x\right)}$$

This form applies to each decision factor for each vehicle and year. “x” is a ratio that indicates the status of that factor relative to the ICEV. “x” can be greater or less than 1. x = 1 means that decision factor has no effect on the purchase decision and $P_{r}=1$. x <1 means the decision factor's status lowers the vehicle purchase probability. x > 1 means the decision factor's status enhances the purchase probability. “a” is a parameter that indicates the importance of the decision factor. a >1 indicates the factor is important and a <1 indicates it is of minor importance. “a” is normally in the range 0.5> a <3. Some of the “x” values are based on detailed calculations of vehicle cost and energy use [39]. For subjective decision factors, the “x” values depend on the judgement of the model user and less quantitative simple calculations. The “a” value for each decision factor depend on the subjective judgement of the model user guided by consumer surveys or market data if they are available (more discussions are shown in section 3.5).

In analyzing the market share for each vehicle type, the value of $P_{r}$ is calculated for each of the decision factors using the input “x” values pertaining to that vehicle. The product ${\pi_{j}}^{n}\;P_{ri}$ of the probabilities $P_{r}$ for all the decision factors is calculated. Herein, *n* represents the total number of decision factors, each associated with a probability. The π symbol is used as a product operator, similar to the summation symbol Σ, but it is used for calculating the product of multiple numbers. This method of calculation is often used to integrate the impact of multiple factors to derive an overall assessment or decision probability. The average decision probability $P_{ravj}$ is determined from the nth root of the product:

(2) $$P_{ravj}={\left({\pi_{j}}^{n}\;P_{ri}\right)}^{i/n}$$

A $P_{ravj}$ value is calculated for each ZEV option (j) being analyzed for a particular vehicle type including the reference ICE vehicle. The market shares of each ZEV option are calculated from:

(3) $$\text{Sum}\left(P_{ravj}\right)=\sum P_{ravj}$$

In modeling vehicle choice for ZEVs, the probability $P_{ravj}$ represents the likelihood of a consumer choosing a specific ZEV option 'j'. Since these probabilities are not inherently normalized, their sum may not equal 1. As market dynamics evolve, the preference for ZEVs may intensify, leading to probabilities that approach 1 for multiple options, causing the cumulative sum to surpass 1. To address this, we normalize the probabilities by ensuring their sum equals 1, which in turn gives us the accurate market share $M_{sj}$ for each ZEV option:

(4) $$M_{sj}=P_{ravj}/\text{sum}\left(P_{ravj}\right)$$

When the sum of ZEV probabilities is less than 1, it implies that there is a remaining market share for ICE vehicles. Hence, the market share for ICE options, $M_{sICE}$, can be computed as:

(5) $$M_{sICE}=1-\;\text{sum}\left(P_{ravj}\right)$$

This scenario reflects a market where ICEVs still have a significant presence, with the market shares for ZEV options given by:

(6) $$M_{sj}=P_{ravj}$$

Conversely, when the sum of ZEV probabilities exceeds 1, it indicates a strong shift towards ZEV options to the extent that ICE options are no longer considered viable alternatives within the model. This leads to a market share of zero for ICE vehicles:

(7) $$M_{sICE}=0$$

The market share analysis is performed using spreadsheets. Calculations are made for several ZEV options – BEV, FC-HEV, FC-PHEV, PHEV, and for an ICE and various LDV types. For LDVs, the vehicle types are mid-size car, small SUV, mid-size SUV, and LD pickup.

The PPA vehicle choice model has been implemented using spreadsheets. The decision factors and associated “x” values are given along with the calculated probability ($P_{r}$) values for each decision factor. The ‘average product’ ($P_{rav}$) and market share ($M_{j}$) for each ZEV option are also shown on the spreadsheets. As for all vehicle choice modeling approaches, there are many inputs needed and some are subjective, non-financial in character. However, for the PPA approach, the inputs and outputs are shown on a single spreadsheet that is easy to follow and the inputs are easy to change. The results are given for each ZEV option, each vehicle type, and each year of the analysis. Consideration of various aspects of applying the DDC-PPA approach to vehicle choice modeling are given in subsequent sections of the paper.

### 3.3 Decision factors

In this section, we examine the decision factors influencing the adoption of ZEVs and the methodology employed to quantify these inputs. Decision factors pivotal to the adoption process can vary widely, encompassing both quantifiable and subjective elements. Financially-oriented factors, such as the initial purchase price, operating and maintenance costs, and potential savings from fuel and tax incentives, are expressed in monetary terms (USD$) herein. These factors play a crucial role in the economic evaluation of ZEV adoption, offering a clear, numerical basis for comparison against conventional vehicles. Conversely, subjective decision factors, which include consumer perceptions of ZEV technology, brand loyalty, environmental consciousness, and the perceived availability and convenience of charging infrastructure, present a challenge for numerical expression. These factors are characterized by personal values, beliefs, and experiences, making them more complex to quantify. Nevertheless, they are integral to understanding consumer behavior and preferences.

#### 3.3.1 Parameter space

(i) Financial decision factors

The decision to purchase a ZEV is significantly influenced by a combination of the initial investment required and the anticipated costs associated with daily and future operations, which include energy consumption and maintenance expenses. Detailed analyses of these costs have been previously conducted by researchers at UC Davis [39,40] providing a solid foundation for understanding the financial considerations of ZEV ownership. The outcomes of these studies are instrumental in establishing the comparative cost ratios between ZEVs and ICE vehicles, which are crucial in assigning values to decision factors 1, 2, 8, 9, and 15 (Table 1). These factors are pivotal in shaping consumer preferences and decision-making processes, particularly in the context of encouraging widespread adoption of ZEVs.

To ensure a robust and forward-looking analysis, the methodology for projecting future costs associated with ZEV ownership is thoroughly detailed in the **Appendices**. This includes an examination of trends in technology advancements, energy prices, regulatory changes, and maintenance requirements that might impact the overall cost of owning and operating a ZEV. By integrating historical data with projections of future trends, this approach offers a comprehensive view of the financial implications of choosing a ZEV over an ICE vehicle.

This understanding of the economic factors influencing ZEV adoption not only aids consumers in making informed decisions but also provides policymakers and industry stakeholders with valuable insights into the barriers and incentives that could affect the pace of ZEV integration into the market. Consequently, the findings from our previous research [[39], [40], [41]] are applied to enhance the precision of the decision-making model, highlighting the critical economic considerations that potential ZEV owners weigh when selecting a vehicle. This integration of empirical research findings into the decision model underscores the importance of economic factors in the transition towards sustainable transportation solutions.

(ii) Non-financial decision factors

The vehicle purchase decision becomes more complex when considering the diverse range of non-financial decision factors that consumers and fleets consider. These factors include the number of available models and brands, the driving experience (such as acceleration and response), fascination with new technologies (such as autonomous driving, electric 4-wheel drive, and large computer screens), environmental considerations, battery durability as well as depreciation and the 2nd market. For ZEVs, driving range and the availability of infrastructure for battery charging [[42], [43], [44]] and hydrogen refueling are crucial decision factors [45,46]. The convenience of battery charging depends both on the availability of chargers and the ability of the battery to accept a fast, complete charge in 20 min or less [[47], [48], [49]]. Refueling hydrogen FCVs can be as fast as ICEVs, but whether it is convenient depends on having an adequate number of hydrogen stations and supply of sufficient hydrogen at a reasonable price. Decision factors 4, 5, and 6 on refueling ZEVs are subjective in character but they have quantitative features that effect the “x” value associated with them. The non-financial decision factors are typically quantified as ‘intangible costs’ in most vehicle choice models and are monetized and then added to the ‘real’ financial costs to determine a lifetime generalized cost of vehicle ownership. Unfortunately, for ZEVs, the intangible costs can be very large and assigning them is critical to the results obtained using the present choice models. It is assumed in the models that customers will purchase the vehicles with the lowest ownership cost and relate the purchase probabilities to the generalized costs. In the PPA model, the average purchase probability is more directly related to the decision factors as shown in Table 1.

#### 3.3.2 The weights of decision factors

Consumer preferences can evolve over time due to technological advancements, fluctuations in fuel prices, new policies, or shifts in societal attitudes. Conducting surveys can aid in determining the weight values of each decision factor for previous years. However, surveys cannot account for the weights in the future market under the highly dynamic nature of the vehicle market. It is crucial to regularly update these factor weights to maintain the relevance and accuracy of the vehicle choice model across the years under different market penetrations of ZEVs. A comprehensive understanding of adoption decisions requires navigating a high-dimensional parameter space that integrates technological, economic, and social or organizational considerations. Therefore, rather than relying solely on surveys of targeted populations, this study sets the weights of decision factors for vehicle choice modeling through a blend of expert judgment, literature review, and model calibration.

California, as a pioneer in transitioning to ZEVs over recent years, has accumulated rich experiences and insights. The summarized consumer preferences and decision factors for vehicle choice in California, drawn from various studies, illuminate diverse aspects influencing the adoption of ZEVs, including plug-in electric vehicles (PEVs) and FCVs. For instance, one study reveals distinct preferences between early adopters and mainstream consumers regarding ZEV attributes [50]. Early adopters show a stronger preference for battery range, acceleration, home charging, and high occupancy vehicle (HOV) lane access. In contrast, mainstream consumers are more concerned with cost-related factors such as fuel and maintenance, as well as charging time. Both groups recognize the importance of monetary incentives, yet they underappreciate the value of public charging station availability. Workshops conducted in California demonstrate the crucial role of PEV owners in informing non-PEV owners about PEVs' benefits and symbolic meanings [51]. This exchange fosters more positive views on PEVs among non-owners, highlighting the significance of community-based education and engagement in promoting PEV adoption. Another paper points out the low PEV adoption rates in disadvantaged communities (DACs), despite available charging infrastructure [52]. It suggests that PEV owners in DACs typically have a higher socioeconomic status than the average community member, identifying barriers to broader adoption such as cost, a lack of awareness about incentives, and access issues to charging facilities for multi-unit housing residents. Further analysis categorizes PEV adopters in California into four distinct groups [53], with high-income families currently leading the market. For continued market growth, it is imperative that middle and mid/high-income groups start adopting PEVs more extensively. Tailoring policies to meet the diverse needs of these groups is essential. The impact of neighborhood and workplace exposure to PEVs on adoption rates is significant [54]. Observing PEVs in residential or workplace settings positively influences PEV sales, though BEV presence might adversely affect PHEV sales due to a substitution effect. This insight calls for targeted policies that take socio-demographic and spatial considerations into account to boost PEV adoption.

In case of FCV market in California, a survey comparing 470 FCV owners with 1550 BEV owners suggests that while there are similarities in gender, education level, household income, and travel patterns between the two groups, notable differences exist in age, ownership history of alternative fuel vehicles, attitudes towards sustainability, and living situations [55]. FCV owners are more likely to live in rented homes and apartment buildings, which may affect their ability to recharge BEVs or install personal chargers. This points towards FCVs potentially appealing to consumers with limited access to private charging infrastructure. However, FCVs face challenges such as a limited number of models and the scarcity of hydrogen refueling stations. Focusing on the role of FCVs in reducing carbon emissions and air pollution, one study examines the governance strategies in California aimed at boosting FCV adoption [56]. Through interviews and document analysis, the study outlines strategies across supply-side, infrastructure, demand-side, and institutional domains, highlighting a blend of strict regulations, incentives, and collaborations between the public and private sectors. Despite these efforts, significant hurdles remain in achieving the desired proliferation of FCVs, mainly due to infrastructural and market challenges. The findings suggest the need for continued innovation in public policy to support the growth of FCVs and hydrogen infrastructure in California and beyond [57].

**Fig. 2:**
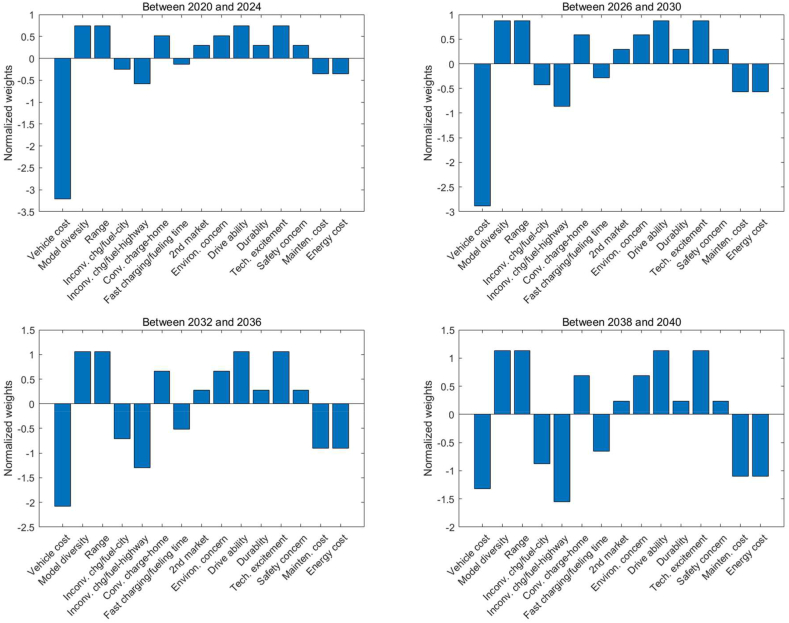
Normalized weights of the decision factors for LDVs.

By integrating the above analysis and insights, Fig. 2 illustrates the normalized weights of decision factors used in this study that influence consumer choices for LDVs across various timeframes: 2020–2024, 2026–2030, 2032–2036, and 2038–2040. These weights underscore the relative significance of each factor within the vehicle purchasing decision-making context of this study. Initially, from 2020 to 2024, the cost of the vehicle exerts the most substantial negative influence. It is posited that for early adopters, the vehicle cost outweighs other disadvantages associated with ZEVs, such as the inconvenience of charging or hydrogen refueling and long charging times. This assessment assumes that early adopters in California may use their primary car for longer trips, while relying on home charging solutions for their everyday BEV use.

As technological advancements and mass manufacturing progress, 'Vehicle cost' becomes less negatively impactful, yet it continues to be a central concern from 2026 to 2030. Progressing to the period between 2032 and 2036, in addition to the vehicle cost, the convenience of highway charging starts to exert a significant negative influence. Moreover, model diversity and range begin to gain increased importance, potentially reflecting a consumer appetite for a more extensive selection of LDV options that cater to varied needs and preferences. Moving into 2038 to 2040, with technological advancements, the cost of vehicles is expected to align more closely with that of ICEVs, thus diminishing its role as a primary factor for consumers. At this point, highway charging, along with energy and maintenance costs, as well as the availability of models and their driving range and capability, ascend in importance for late adopters. This evolution signals a consumer demand for a wider variety of models and an elevated consciousness of the operating costs associated with LDVs.

Overall, the normalized weights illustrate a dynamic market in which consumer priorities shift in response to technological advancements, market developments, and potential policy changes. The growing emphasis on model diversity and rapid charging in later years points to a maturing market where consumer interests go beyond basic availability to specific features that enhance the driving experience and convenience. The consistently positive weight of environmental concerns suggests a durable or growing market segment that prioritizes environmental impact in their purchasing decisions.

### 3.4 Preparation of the input data for various ZEV development scenarios

**Fig. 3:**
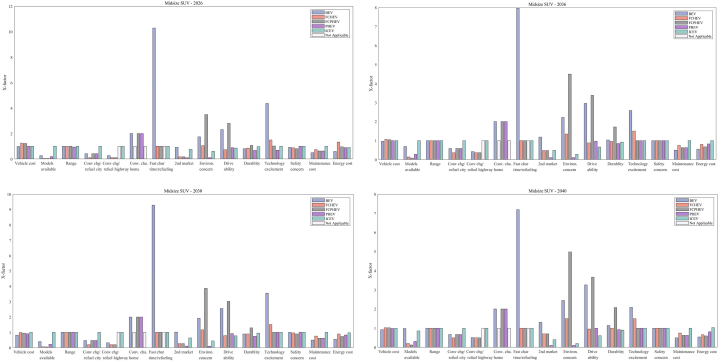
Some x-factor values for vehicle choice modeling.

The key step in applying the PPA model is setting up the base and “x” value tables for each ZEV and ICEV to be considered in the market analysis. The tables will include values for the time period 2020–2040. During this period, the vehicle technologies, the ZEVs on the market, and the refueling infrastructures will be maturing simultaneously. Hence the tables must reflect the effect of radical changes in all these areas, which will be affected by industrial strategies and public policies. This results in the need to consider multiple market penetration scenarios. The “x” values for LDV modeling and how they change in future years are shown in Fig. 3.

The decision factors and how the “x” values are determined are discussed in detail in **Appendices.** Brief discussions of the key decision factors shown in Fig. 1 are given in the following paragraphs.

#### 3.4.1 LDVs

(i) **Vehicle Cost:** During the early to mid-2020s, the high costs of battery-electric LDVs were a significant barrier to their widespread adoption. These high prices were chiefly attributed to the expenses related to the production of batteries/fuel cells and the implementation of innovative manufacturing technologies. R&D advancements in ZEV production and manufacturing processes resulted in steady reductions in the cost of BEVs. The cost projections in Ref. [39] indicate that the cost of LD BEVs will be slightly less than their ICEV counterparts by 2030, while the cost of FCVs are expected to close to that of ICEVs after 2030. Enhancements in economies of scale, further development in battery and fuel cell technologies, along with the maturation of the EV supply chain, are needed to achieve the projected decreases in the cost of ZEVs. Comparisons of the UCD projections [43] with cost projections from other groups are discussed in Appendix B**.**
(ii) **Model Available:** Throughout the early to mid-2020s, the variety of ZEV models available are expected to be limited. Despite a growing number of automakers investing in ZEVs, the selection will still be modest compared to conventional vehicles especially for lower priced BEVs. The number of models of FCVs will grow slowly due to an immature hydrogen infrastructure and high FC vehicle costs. By 2040, it's anticipated that nearly all automakers will offer a comprehensive portfolio of ZEVs, particularly a broad range of BEVs. This transformation is projected to be fueled by rising consumer demand, tighter regulatory requirements, and substantial technological progress. Consequently, consumers will have a wider array of choices.
(iii) **Inconvenience of Charging/Fueling in the City:** At the onset of the 2020s, users of city-based ZEV LDVs, particularly BEVs and FCVs, will encounter significant obstacles due to the lack of charging and refueling infrastructure. Some owners of BEVs will have home charging and only a limited need for public chargers. In the case of FCVs, while boasting quick refueling times akin to conventional gasoline vehicles, they will be totally dependent on public hydrogen refueling stations which will be limited in number until 2030. By 2040, it's anticipated that there will be adequate numbers of both public charging and hydrogen refueling stations in cities/towns and refueling for both BEVs and FCVs will be convenient.
(iv) **Inconvenience of Charging/Fueling on the Highway:** Throughout the 2020s, the scarcity of charging and hydrogen refueling stations along highways will pose significant hurdles for long-distance travel with ZEVs. This deficiency will be especially annoying to drivers of BEVs because of the time required to charge the battery. It is expected that all charging stations on the highway will have high power chargers and the charging times will decrease to 10–15 min [47] by 2030. FCVs, while benefiting from longer ranges and faster refueling times, were still be constrained by the limited availability of hydrogen refueling stations until along some highways until after 2030. However, by 2040, it is anticipated that a comprehensive battery charging and hydrogen refueling infrastructure will be established along all major highways.
(i) **Safety Concerns:** During the 2020s, there will be some safety concerns in relation to the handling and storage of large-kWh batteries for BEVs and the onboard storage of large kg of hydrogen for FCVs. The largest safety concern will be vehicle fires due to battery failures [58,59]. The possibility of failure will increase with the size of the battery containing many more cells and the fires when they occur will be much larger and more difficult to extinguish [[60], [61], [62]]. Hence monitoring the condition of the battery and battery safety in general will be more important for M/HDVs than for LDVs [[63], [64], [65]]. In addition, there will be some uncertainties about the performance of the ZEV M/HDVs in commercial operating conditions. As more ZEV trucks of various classes are in operation, all these factors must be monitored carefully because extensive use of electricity and hydrogen in ground transportation will be a new experience. By 2030, it's anticipated that the safety uncertainties will be greatly reduced and safe operation of ZEVs will be expected and well regulated.

#### 3.4.2 Input scenarios

In this study, eight scenarios are defined to evaluate the impact of various decision factors on the market share of ZEVs. The principal differences between the scenarios are the rates at which the battery charging and hydrogen refueling infrastructures are expanded and the degree that the costs of the batteries and fuel cells are reduced by the manufacturers. It will be assumed that the present rapid development of batteries and fuel cells will continue resulting in the significant decrease in their cost. Calculations of the projected market penetration of ZEVs will also be made with and without incentives to reduce their purchase cost. The expansion of the infrastructures and vehicle cost incentives will depend on both federal and California government policies.

The various scenario cases for LDVs, Sxyz, are defined in Table 2 where x (1–3) designates the infrastructure expansion condition for the scenario, y (1–3) designates the cost (high, base, and low) of the battery and fuel cell components, and z designates the value of incentives (1 or 2). This same code is used to mark the curves in the presentation of results in all the figures in this study.

**Table 2:** Vehicle penetration scenarios under different assumptions for LDVs.

Model	Code	Battery cost	Fuel cell cost	Chargers+/inconvenience	H2 stations+/inconvenience	Incentives
S1	S111	High	High	Base Charger availability	Base H2 availability	With^a^
	S121	Base	Base	Base Charger availability	Base H2 availability	With^a^
	S131	Low	Low	Base Charger availability	Base H2 availability	With^a^
	S122	Base	Base	Base Charger availability	Base Chargers availability	With^b^
S2	S221	Base	Base	Expanding Charger availability	Base H2 availability	With^a^
S3	S321	Base	Base	Base Charger availability	Expanding the H2 availability	With^a^
S4	S431	Low	Low	Expanding Charger availability	Expanding the H2 availability	With^a^
	S432	Low	Low	Expanding Charger availability	Expanding the H2 availability	With^b^

a Incentives and rebates including Clean Vehicle Tax Credits (CVTC) [66] between 2020 and 2032 and the Clean Vehicle Rebate Program (CVRP) [67] between 2020 and 2022.

b Both CVTC and CVRP are available between 2020 and 2040, but with linear decrease year by year since 2032.

Scenario 1 (S1) is the base scenario and considered the most likely unless strong government policies are in place to encourage sales of ZEVs. It includes four different scenarios, including S111, S121, S131 and S122. The first three is used to investigate the effect of vehicle costs on the market share of ZEVs, including high, base, and low costs of energy storage and power production devices (i.e., batteries and fuel cells) under different technology improvements. Although the costs of BEVs are currently much higher than those of comparable ICE vehicles, the price difference is expected to decrease by 2030 due to the marked decrease in the cost ($/kWh) of batteries over the past decade. Fuel cells are presently costly ($/kW), and whether their cost will decrease markedly as batteries have depends on how rapidly the market for them develops. The projected cost of batteries and fuel cells are discussed in Appendix B. The last one, S_122_ is used to investigate the effect of financial incentives on the market share of ZEVs for the base costs of batteries and fuel cells.

Scenario 2 (S2) examines the business-*as*-usual scenario with BEVs dominating and a government-supported, reasonably sized battery charging infrastructure.

Scenario 3 (S3) is designed to reflect the rapid expansion of the hydrogen infrastructure and the gradual response of vehicle manufacturers to provide FCVs for sale.

Scenario 4 (S4) includes two cases – with incentives by 2032 and by 2040 under low costs of batteries and fuel cells and sufficient charging and hydrogen refueling infrastructure by 2040, reaching similar convenience level (refueling in 20 min or less) as ICE vehicles. Various aspects of providing the infrastructure for ZEVs are discussed in Appendix A.4.

Overall, these scenarios aim to provide insight into the effects of various factors on the market share of ZEVs in California, including vehicle costs, financial incentives, battery charging and hydrogen refueling infrastructure as well model availability.

### 3.5 Calibration of the model

Model calibration refers to the process of adjusting the output of a model so that it reflects the probabilities of the outcomes it is modeling. In other words, a calibrated model is one that produces predicts probabilities that are close to the actual probabilities of the events it predicts. Calibration is important because it shows that the inputs to the model can be adjusted to attain results in good agreement with actual data for market shares for BEVs sold in 2020 and 2022 [68]. In this study, the primary data inputs for model development and calibration encompass a diverse range of information, essential for a comprehensive analysis. These are based on the statistics provided by the CEC [69]: (1) California Market Data (2020–2022): This crucial data set provides detailed insights into the manufacturers, volume of vehicle sales, and various vehicle characteristics, offering a robust foundation for the study. (2) Technology Data: This encompasses current information on ZEV technologies, including data on vehicles purchased, their manufacturer suggested retail prices (MSRP), and for used vehicles, details on vehicle miles traveled are also included. Additionally, the data covers fuel efficiency metrics for both ZEVs and ICEVs. (3) Infrastructure Details: Information on the availability and distribution of charging and hydrogen stations is a key component, reflecting the infrastructure's role in supporting ZEV adoption. (4) Government Incentives and Policies: This includes a comprehensive overview of incentives and policies implemented by the California government and at the federal level, which are instrumental in shaping the market dynamics for ZEVs. Together, these data inputs provide a multi-dimensional perspective crucial for understanding and analyzing the market trends, technological advancements, and policy impacts in the context of ZEVs. Herein, it involves the preparation of the probability tables for the BEV and FC-HEV for 2020 and 2022 and comparing the calculated results with the actual market data. The comparisons of the PPA results and market data for LDVs show that the PPA model can be used to make reasonable market share projections.

## 4 Results obtained using the PPA model for different scenarios

### 4.1 Light-duty vehicle markets

**Fig. 4:**
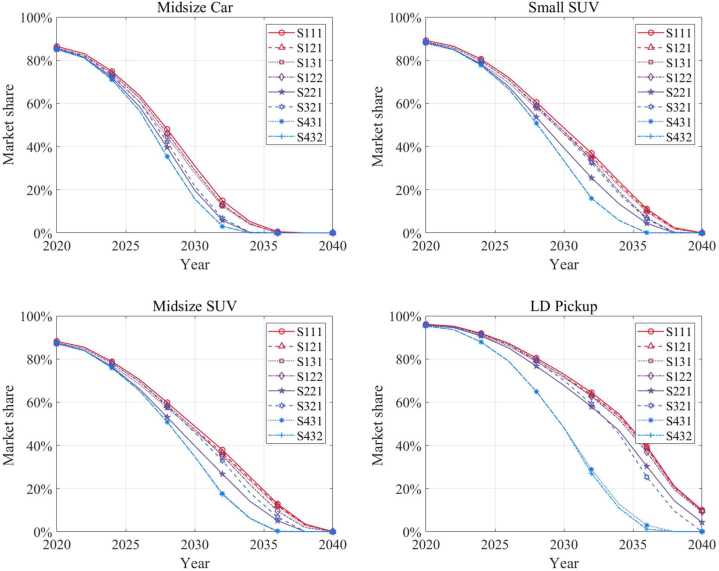
Projected market shares of light-duty ICEVs in the California for all the scenarios.

To achieve the net-zero GHG emissions goal in California by 2045, ZEVs (BEVs, FCVs, and PHEVs) must completely dominate the on-road vehicle stock. This requires the annual sales of ZEVs to dominate the market well before 2040. Therefore, the growth in sales share of ZEVs over the next two decades is crucial for transitioning to cleaner transportation energy sources, provided that grid decarbonization is happening concurrently. Fig. 4 illustrates the market dynamics of eight scenarios (defined in Table 2), depicting the decline of sales of ICEVs. The findings reveal a rapid expansion of ZEV sales across some scenarios. In the midsize car segment, the decline is pronounced and uniform across all scenarios, suggesting a consistent trend towards obsolescence of ICEVs in this category. By approximately 2035, the market share of ICEVs dips below 20 % for most scenarios, indicating a significant shift in consumer preference or policy influence favoring ZEVs. Similarly, the small SUV segment exhibits a comparable decline, albeit with a slight variation among scenarios in the latter years approaching 2040. This could imply potential differences in regulatory impacts, technological advancements, or market forces that are scenario-dependent. The midsize SUV and light-duty pickup categories show a steeper initial decline followed by a gradual plateau as the market share approaches the 20 % threshold. This plateauing effect could be indicative of a persistent specialized market for ICEVs or a slower adoption rate of alternative fuel vehicles in these segments. The convergence of scenarios around the year 2035 for all vehicle types could be reflective of a pivotal policy intervention or a technological breakthrough that accelerates the decline of ICEVs. This alignment of market trends across different vehicle categories underscores the potential influence of statewide or nationwide factors over the automotive market.

**Fig. 5:**
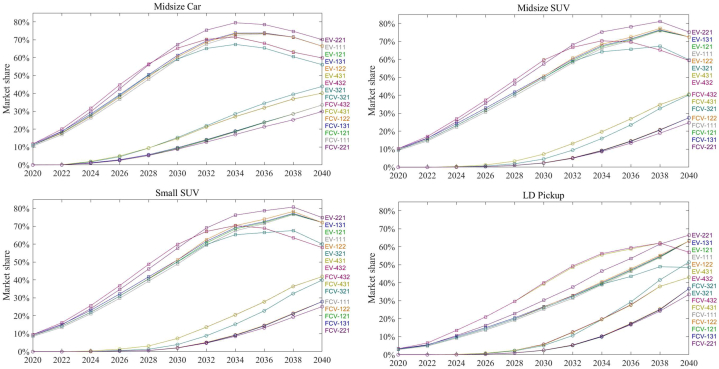
Projected market shares of light-duty EVs (BEVs & PHEVs) and FCVs (FCHEVs & FCPHEVs) in the California for all the scenarios.

Fig. 5 shows the market penetration of EV (BEV & PHEV) and FCV (FCHEV & FCPHEV), respectively. The findings reveal a rapid expansion of ZEV sales across some scenarios. In this study, ICEVs include both gasoline-powered and non-plugin hybrid vehicles. A closer examination of each graph reveals the market penetration trends for EVs and FCVs from 2020 to 2040 under various scenarios. Across all vehicle categories, EVs demonstrate an upward trajectory in market share, peaking around the 2030 to 2035 timeframe before stabilizing or slightly retracting. The BEVs, particularly in the scenarios EV-221, EV-121, EV-321, and EV-431, display a robust ascent, underscoring the growing consumer acceptance and likely improvements in battery technology and charging infrastructure. The market share of PHEVs, though increasing, appears to follow a more modest curve compared to BEVs, potentially reflecting consumer preferences for the latter's pure electric driving experience and the maturation of electric charging infrastructure that diminishes the range anxiety associated with earlier EV models. FCVs, including both FCHEVs and FCPHEVs, show a more gradual and steadier climb in market share, with some scenarios (FCV-321, FCV-431) suggesting a more optimistic outlook. The FCV trends might be indicative of advancements in hydrogen fuel cell technology, infrastructure development, and possibly policy incentives that make FCVs a more viable option over time. Distinctively, the LD Pickup category exhibits a different pattern with FCVs gaining a significant market share compared to other vehicle types. This could be attributed to the specific use-case scenarios of pickups where the longer range and quick refueling of FCVs might be particularly advantageous. Overall, Fig. 5 provides a comprehensive outlook on the light-duty ZEVs in California, portraying a significant increase in market shares across all scenarios. This section will focus on each scenario and provide analysis of the results.

#### 4.1.1 Scenario_1

This is the base case for the 3 levels (high, base, and low) of battery and fuel cell costs between 2020 and 2040. For BEVs, the charging availability – the convenience in the city and on the highway – is assumed to be 0.33–0.66 (city) and 0.2–0.5 (Highway), respectively, between 2020 and 2040. For PHEVs, we assume that the owners only consider the inconvenience of charging in the city, which is set to be equal to the case of BEVs. For FCVs, the hydrogen station availability – the convenience in the city and on the highway – is assumed to be 0.005–0.16 between 2020 and 2040. For FCPHEVs, the vehicle owners will charge their vehicles primarily in the city, which is assumed to be equal to the case of BEVs and PHEVs, while they will refuel primarily with hydrogen on the highway, which is assumed to be equal to the case of FCHEVs. The model results suggest that variations in battery and fuel cell costs within the range considered in this study will not significantly impact the market share of ZEVs, including both EVs and FCVs, by 2040 (less than 3 % across the years).

**Fig. 6:**
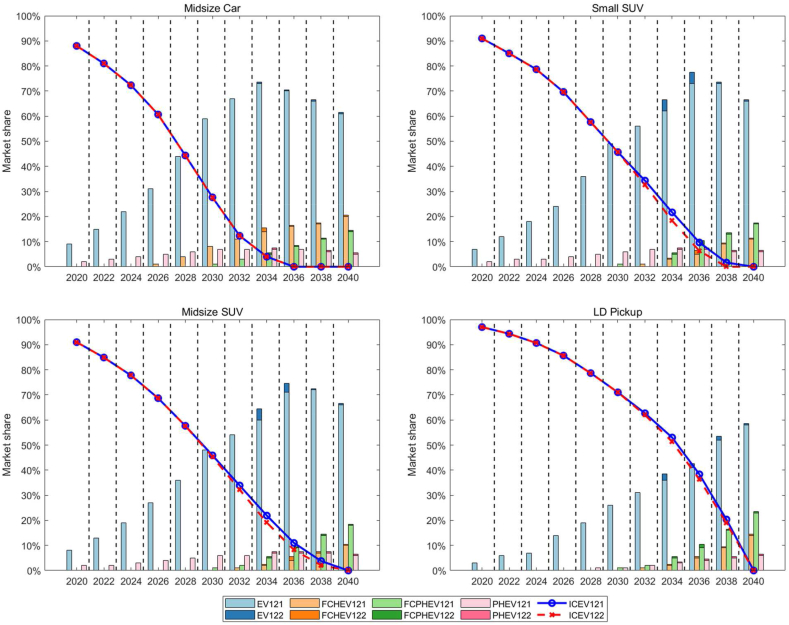
Market share of ZEVs under different financial incentive plans for the S1 infrastructure cases (S121 and S122).

Beyond examining the effect of vehicle cost on the market penetration of ZEVs, Scenario 1 also investigates the impact of financial incentives (Appendix B.4) on the market share of ZEVs, as depicted in Fig. 6. This study explores the impact of financial incentives based on current policies in California, such as Clean Vehicle Tax Credits and the Clean Vehicle Rebate Program for LDVs, with a particular focus on their continued influence between 2034 and 2040. The findings suggest that even if financial incentives for purchasing ZEVs are discontinued, the market share of ICEVs would see only a minor decline.

In the LDV market, various financial incentives explored in this study did not substantially affect ICE sales. This outcome appears to be due to the prices of these vehicles, which are nearing equivalence with ICE counterparts. Consequently, the absence of financial incentives post-2036 for midsize vehicles is unlikely to have a significant impact.

Interestingly, the market share of FCVs, including FCHEVs and FCPHEVs, does not show sensitivity to financial incentives. This could be attributed to the limited availability of vehicle models and the inadequacy of hydrogen refueling infrastructure. Thus, implementing financial incentives solely for FCVs is unlikely to have a major influence on sales until the availability of more models increases and the hydrogen infrastructure undergoes significant improvement. However, it is worth noting that if both Clean Vehicle Tax Credits and Clean Vehicle Rebate Program incentives are sustained until 2034 for midsize cars, near 100 % market penetration for ZEVs appears achievable by 2034.

#### 4.1.2 Scenario_2

This scenario is based on the base case for the cost of battery-electric and fuel cell components and an improved battery charging infrastructure. The scenario assumes that by 2040, DC fast chargers, both in the city and along highways, will offer a level of convenience comparable to that of gasoline ICE refueling, with charging times of 20 min or less. The battery charging infrastructure will improve gradually between 2020 and 2040. In this scenario, it is assumed the infrastructure for FCVs is changed little from S1.

**Fig. 7:**
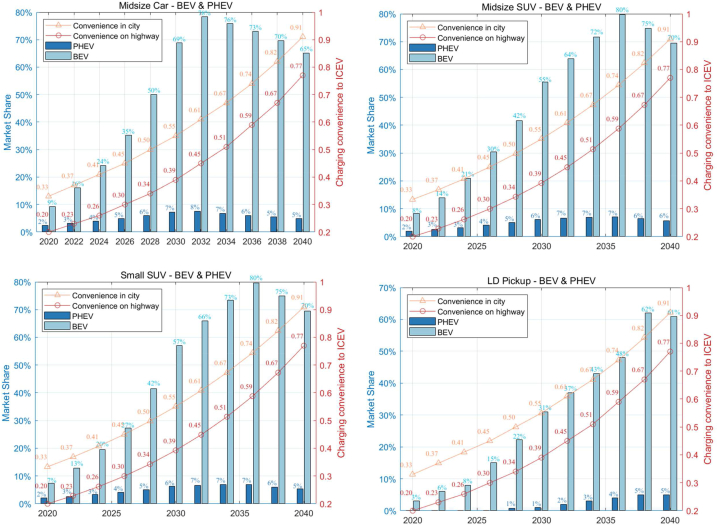
Market shares of BEV and PHEV under improved charging infrastructure for S2 (S221).

Fig. 7 illustrates the growing market penetration of BEVs and PHEVs across various LDV segments under Scenario 2 (S221), where a significant enhancement in EV charging infrastructure is anticipated. The projections indicate that by 2040, BEVs will achieve over 50 % market share in the Midsize Car and Small SUV categories, pointing to a decisive consumer shift towards electric mobility. This trend is underpinned by the expected improvements in charging infrastructure, which are predicted to alleviate concerns about range and charging times, contributing to the appeal of BEVs. While PHEVs also gain market share, they lag behind BEVs, suggesting a preference for the latter's operational efficiency. The uptake of BEVs in the Midsize SUV and LD Pickup sectors follows a similar upward trajectory, although at a slower rate for LD Pickups, potentially due to their specific performance requirements. The projection of charging convenience nearing the ease of traditional ICEV refueling by 2040 further cements the likelihood of EVs becoming the vehicles of choice, driven by infrastructure developments that promise to ease the transition to electric mobility.

**Fig. 8:**
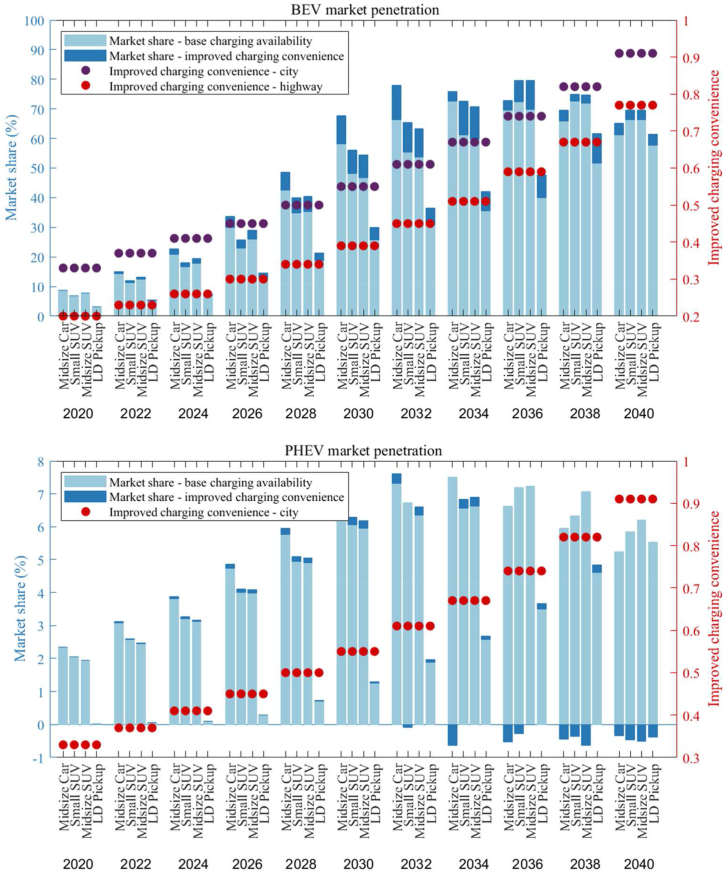
Market shares of BEV and PHEV under different charging availability (S121 and S221).

Fig. 8 offers a comparative analysis of the market shares of BEVs and PHEVs under varying conditions of charging availability. It underscores the significant influence that enhanced charging infrastructure has on the market prevalence of these vehicles. The dual scenario analysis contrasts the baseline charging availability (S121) with improved charging convenience (S221), showcasing the correlation between charging infrastructure and vehicle adoption rates. The bar charts articulate a clear trend: with better charging infrastructure, the market share of BEVs is projected to increase significantly. In specific vehicle categories, this surge exceeds 20 %, which is a substantial margin considering the breadth of the automotive market. The data suggests that the market share of PHEVs is likely to diminish in scenarios where BEV charging infrastructure is augmented, reinforcing the hypothesis that consumers have a marked preference for BEVs when charging convenience aligns more closely with the traditional refueling experience. The detailed overlay of improved charging convenience, plotted with dots for both city and highway contexts, reveals that consumer confidence in BEVs correlates strongly with the accessibility and speed of charging. As charging times decrease and become more widely available, the convenience factor appears to play a critical role in tilting consumer preferences towards BEVs, a pattern observable across the two-decade span from 2020 to 2040.

#### 4.1.3 Scenario_3

In S3, it is assumed that the hydrogen refueling infrastructure will be improved to the same convenience of refueling as gasoline ICEVs at both highway and city stations by 2040. Availability rates for the intervening years will be linearly interpolated. The base cost of fuel cells is used in this scenario and it is assumed that more FCV models are available for purchase with improved hydrogen infrastructure.

**Fig. 9:**
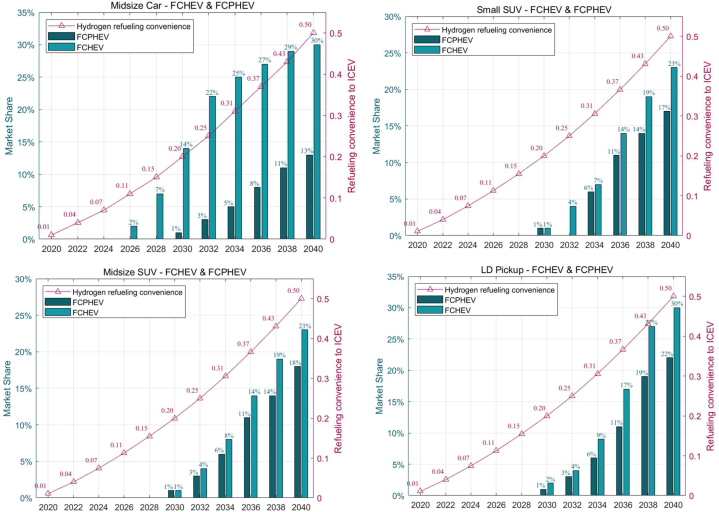
Market shares of FCHEV and FCPHEV under improved hydrogen refueling infrastructure (S321).

Fig. 9 presents a forward-looking depiction for FC-HEVs and FC-PHEVs as delineated in Scenario 3 (S321). This scenario is predicated on the enhancement of hydrogen refueling infrastructure to match the convenience of gasoline ICE vehicle refueling by the year 2040. In all categories, FCVs are expected to achieve around 30 % market share by 2040, a substantial representation indicative of robust growth in the FCV market. The climb in market share is coupled with a corresponding rise in perceived refueling convenience (red line), which suggests a strong positive correlation between consumer adoption of FCVs and the availability and practicality of hydrogen refueling stations. The data provides empirical insights into how infrastructure can be as crucial as vehicle technology advancements in driving market shifts. The non-linear interpolation of availability rates for intervening years emphasizes the presumed gradual but consistent improvement in refueling infrastructure. Additionally, the projection that more FCV models will become available aligns with a key premise of diffusion of innovations theory, which posits that the diversity and availability of options are critical to the uptake of new technologies. The graph indicates favorable conditions for the widespread adoption of FCVs, as both vehicle technology and infrastructure are advancing simultaneously.

**Fig. 10:**
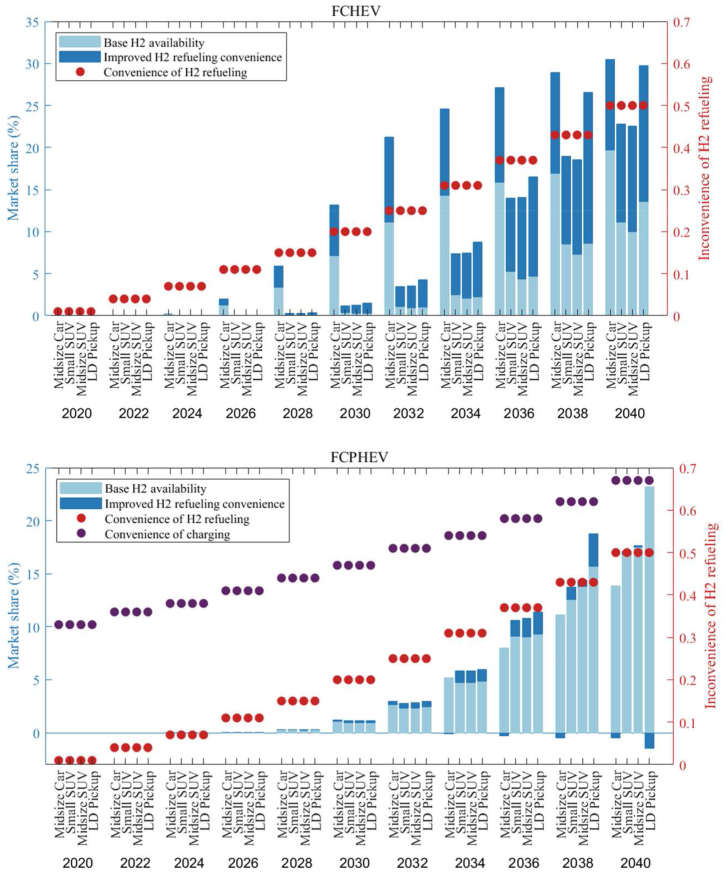
Market shares of FCVs (FC-HEV & FC-PHEV) under different H2 availability (S121 and S321).

Fig. 10 presents a comparison between the base case and an improved scenario for hydrogen availability. It demonstrates that enhancing the availability of hydrogen refueling stations has a significant impact on the market share of FCVs by 2040. The light blue bars represent the base hydrogen (H2) availability scenario, while the darker blue bars indicate the scenario with improved H2 refueling convenience. The red dots correspond to the convenience of H2 refueling, and the purple dots reflect the convenience of charging. In the improved scenario, the market share of FCHEVs exhibits a marked increase, especially after 2030, indicating that enhanced hydrogen infrastructure is a crucial driver for the adoption of these vehicles. By 2040, the market share reaches a notable level, suggesting that consumers may be highly responsive to the improved refueling infrastructure. However, although the market share for FCPHEVs is generally positively influenced by the improved H2 refueling scenario, some specific segments may experience a slight decline, such as in 2040, if hydrogen refueling meets basic daily consumer needs at a convenience level that is half as convenient as that of ICEVs.

#### 4.1.4 Scenario_4

**Fig. 11:**
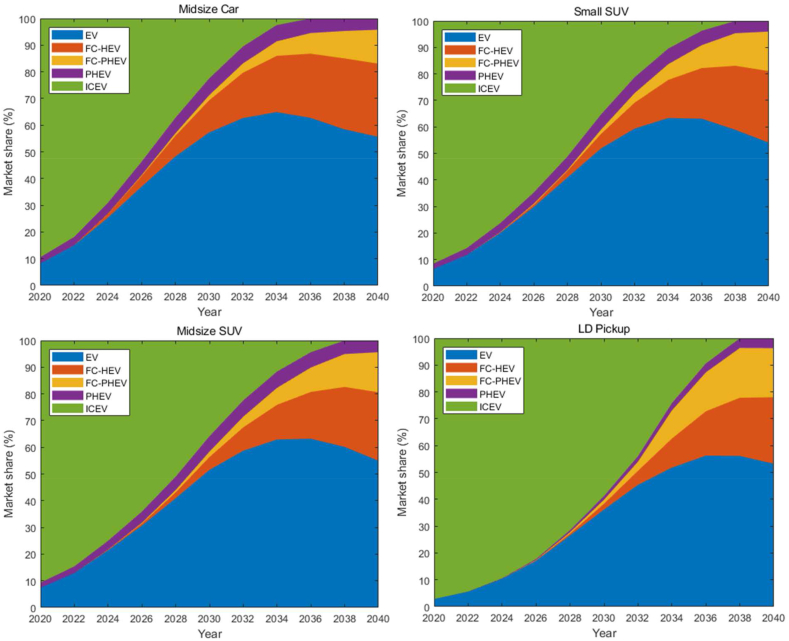
Market shares of ZEVs in California for the S4 scenario.

This scenario explores market conditions needed to achieve the CARB targets for the sale of ZEVs — that is, 2025: 10 % of new passenger car sales to be ZEV, 2030: 55 % of new passenger car sales to be ZEVs, 2035: 100 % of new passenger car sales to be ZEVs. The PPA model results for the S4 scenarios are shown in Fig. 11. In short, California can achieve 100 % ZEV sales by 2035 for LDV. Specifically, In the earlier years, ICEVs occupy the largest market share, which begins to shrink steadily as the market share of ZEVs grows. The transition appears to be led by BEVs, which show a significant increase in market share as early as the 2020s, suggesting rapid adoption and market penetration. FC-HEVs and FC-PHEVs also expand their market presence, but their growth is more gradual compared to EVs, potentially reflecting the current state of hydrogen fuel infrastructure and technology. PHEVs maintain a consistent presence in the market, serving as a transitional technology before the complete switch to full ZEVs. By 2035, the CARB target of 100 % new passenger car sales being ZEVs is achieved across all categories, evidenced by the complete phase-out of the ICEV area in the chart. This indicates that policy measures and technological advancements are expected to work in tandem to achieve the ambitious decarbonization goals set by the state. The LD Pickup category is highlighted as the most challenging segment for achieving the ZEV transition in the early years. In order to meet 100 % ZEV sales by 2036, financial incentives seem to be needed. The output of S431 shows that the 100 % ZEV target can be achieved before 2036 if the Clean Vehicle Tax Credits are available until 2032. In addition, the low costs of batteries and fuel cells are needed as well as the aggressive construction of charging and hydrogen refueling infrastructure over the next two decades.

### 4.2 The sensitivity analysis of the decision factors

Sensitivity analysis of the vehicle choice model involves examining how changes in input parameters (decision factors) affect the model's outputs (market share). It involves systematically varying the inputs assigned to each decision factor to assess the impact on the market share predictions for ZEVs and ICEVs. This analysis is essential for identifying which factors significantly influence the adoption of ZEVs, thereby providing valuable insights for policymakers and manufacturers in shaping strategies to promote ZEV uptake.

**Fig. 12:**
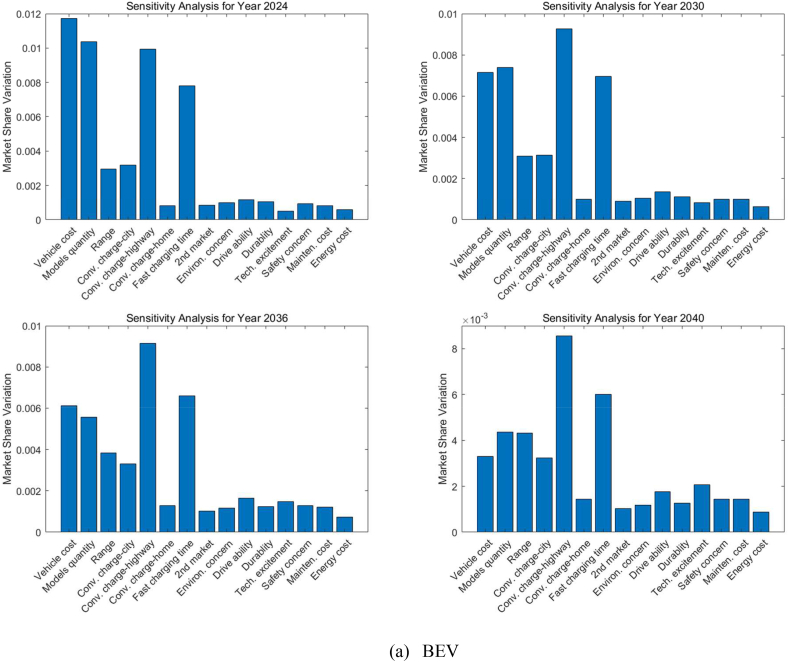

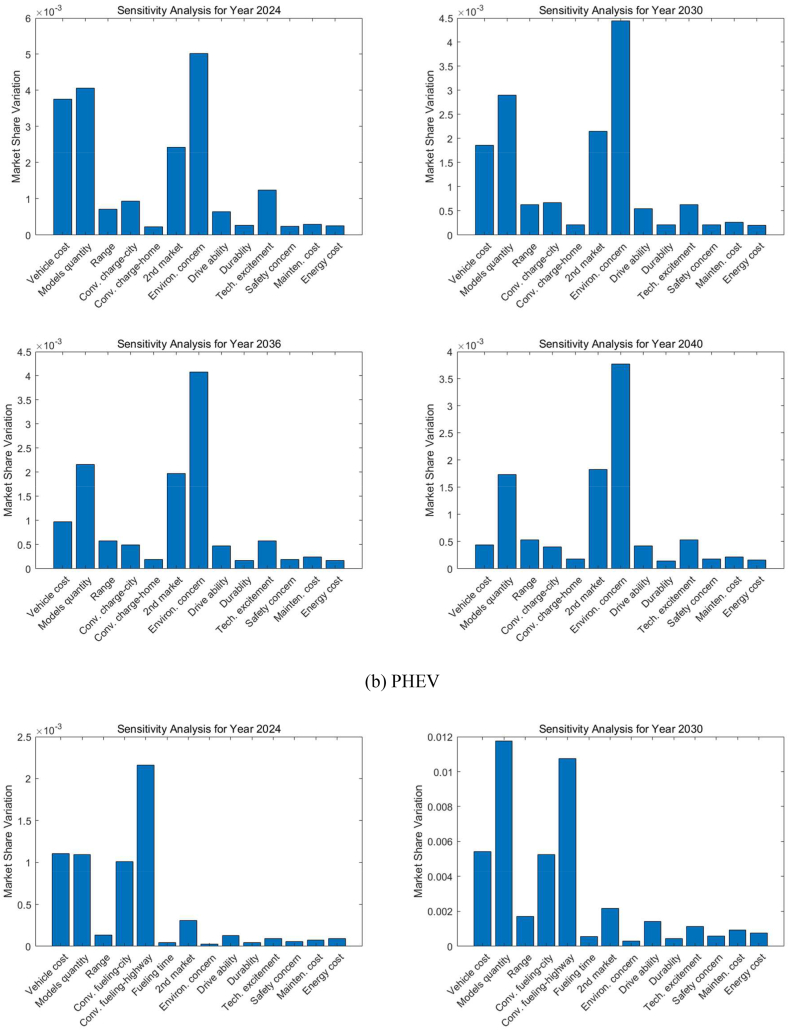

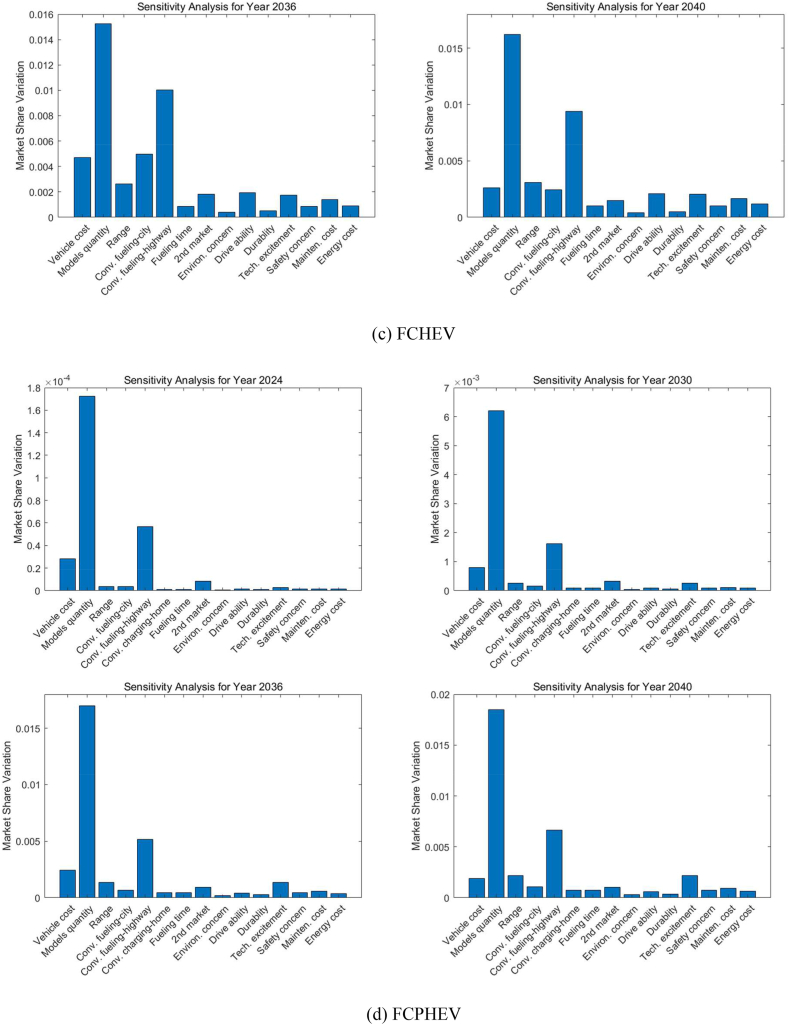
Sensitivity analysis of the decision factors for LDVs (Midsize car as an example).

Fig. 12 represents the sensitivity analysis for different ZEV technologies in various forecast years. The sensitivity analysis evaluates how sensitive the market share is to changes in input parameters, indicating which factors have the most impact on market share variations for LDVs.

As shown in Fig. 12(a), for BEVs in 2024, the attributes with the highest sensitivity are related to vehicle cost, model diversity, and the convenience of charging on the highway, as well as fast charging time. These factors suggest that even minor improvements could significantly boost the BEV market share, highlighting areas for focus such as initial purchase price, battery technology, and public infrastructure development. As we progress to 2030, all the important decision factors show decreased sensitivity but still dominate the market penetration of BEVs. By 2036, the convenience of charging on the highway becomes the foremost factor, signifying that the ease of public charging continues to be a pivotal influence on the adoption of BEVs. Along with infrastructure availability, charging time occupies the second most important position in influencing market variations among all decision factors. By 2040, similar factors to those important in 2036 remain significant; however, vehicle cost emerges as a less sensitive factor due to technological improvements and BEV costs approaching those of ICEVs.

The analysis in Fig. 12(b) shows that environmental concerns greatly influence how consumers feel about PHEVs for the next twenty years. While the concern over how much a vehicle costs is becoming less important over time, many people remain unsure about whether PHEVs really help the environment. This makes them less popular in California, despite the fact that PHEVs address worries about running out of battery and the need for frequent recharging on long trips thanks to their gas engine. California is well-known for its strong environmental policies and support for adopting green technologies, and its residents place a high value on how their vehicle choices affect the environment. BEVs, which don't emit any pollutants while being driven on electricity, are often seen as better for the environment than PHEVs. This is because PHEVs can still produce emissions when they switch to using their gas engine. This difference might make consumers believe that BEVs do more for environmental protection than PHEVs, leading them to prefer BEVs, especially in a state that's serious about cutting down on car emissions.

For FCHEVs, shown in Fig. 12(c) in 2024, the ease of hydrogen refueling (highway) is highlighted as the most critical factor for FCHEVs, with the initial purchase price also presenting a significant obstacle to consumer adoption. While this concern lessens by 2030, it remains a notable issue, indicating that the cost will persist as a decisive factor for prospective buyers. The market's responsiveness to FCHEVs will be significantly influenced by the variety of available models and the convenience of refueling by 2030. The presence of hydrogen refueling stations is identified as a crucial element in the FCHEV market for the forthcoming decade. By 2036, the diversity of models stands out as the factor with the highest sensitivity, emphasizing the need for a broad selection of models and strong branding to achieve widespread adoption of FCHEVs. Moving to 2040, both model diversity and the expansion of hydrogen infrastructure, particularly along highways, continue to be predominant factors. These points of sensitivity reflect consumer expectations for the convenience of refueling during long trips and a preference for a wide range of models.

In the case of FCPHEVs, as Fig. 12(d) shows, 'Model diversity' remains the leading sensitive factor for the next two decades, reinforcing the idea that consumer preference for a range of vehicle models is crucial in the decision-making process for potential buyers. This suggests that an expanded range of model options could significantly increase market share. Since FCPHEVs can potentially be charged at home or work, the next important factor is the convenience of hydrogen refueling on the highway. The sensitivity analysis also shows that before a variety of FCPHEV models become available and a basic hydrogen refueling infrastructure is established, all other factors will not significantly influence the market penetration of FCPHEVs through to 2040.

The evolution of these sensitivities across different powertrain technologies over time suggests that as the market matures, consumer expectations evolve, highlighting the need for the industry to adapt to these changing demands. These insights are vital for manufacturers, policymakers, and infrastructure planners to prioritize investments and policy measures that are in tune with these sensitivities, thereby fostering the growth of the LDV market.

### 4.3 Comparison of the results between this study and models developed at the DOE National Laboratories

The DOE National Laboratories have been developing for a number of years methods/models to project ZEV market shares during 2020–2050. It is of interest to compare the ITS-UCD results with the DOE projections from their papers. DOE Oak Ridge/Argonne Labs have made projections for LDVs. We obtained the MA3T program from Oak Ridge Lab and have run the program to obtain results for California to compare with results using the PPA model.

As discussed in Section 3, the PPA approach deals directly with the purchase probabilities associated with each of the decision factors and calculates an average probability for each vehicle option. A market share for the vehicle options is determined from the average probabilities. All the vehicle choice models must cope with non-financial factors which require subjective judgements to include in the model. In addition, the inputs to the models are expected to change as the ZEV technologies mature over the next 20+ years. How these technologies and the associated vehicle infrastructure develop are uncertain and the subject of much speculation. As a result, all the groups doing vehicle choice modeling construct different scenarios on which their market share results depend. In making our comparisons of results from the different models, we will indicate key factors and differences in the scenarios for which the results apply.

The LDV comparisons will be conducted using results from the MA3T program at Oak Ridge/Argonne Labs. We will incorporate recent findings from Ref. [12] and a specific run conducted at UC Davis. Additional MA3T results are provided in Ref. [10]. Notably, the most recent MA3T results do not indicate a market for fuel cell powered LDVs. The data presented in Table 3 focuses exclusively on BEV and PHEV plug-in vehicle options. The scenarios analyzed are as follows: S1- base battery costs with limited battery charging infrastructure, S2- lowest battery costs with minor improvements in battery charging, and S4- lowest battery costs with optimal battery charging. According to the results in Table 3, enhancements in battery cost and infrastructure lead to increased market shares for plug-in vehicles, with PHEVs showing the most significant gains in the later years. These findings suggest a market resistance to BEVs once their sales share reaches approximately 40 %. The MA3T results in Table 3 are assumed to be representative of the United States as a whole.

**Table 3:** MA3T LDV results for the United States for various battery scenarios [12].

Year	BEV-S1 Base battery cost Base battery charging facility	PHEV-S1 Base battery cost Base battery charging facility	BEV- S2 Base battery cost Expanding Charger availability	PHEV-S2 Base battery cost Expanding Charger availability	BEV-S4 Optimized battery cost Expanding Charger availability	PHEV-S4 Optimized battery cost Expanding Charger availability
2025	8	3	8	3	8	3
2030	12	8	25	5	32	7
2035	22	6	34	12	42	16
2040	26	12	34	18	42	28
2045	28	14	33	26	41	39
2050	30	20	32	36	41	49

The MA3T program was run at UC Davis using the inputs as received from Oak Ridge Lab. The results shown in Table 4 are for California. This version of the MA3T inputs resulted in significant sales of fuel cell powered vehicles, but nearly all those vehicles were FCPHEVs. The results in Table 4 indicate a very rapid increase in market share for BEVs up to 2030 and a slower decrease in BEV market share as the PHEV and FC-PHEV market shares increase in later years beyond 2030. This trend is consistent with the results shown in Table 3.

**Table 4:** MA3T LDV results for California.

Year	Total sales (k)	% BEV	%FC-PHEV	%PHEV	Total % ZEV
2020	784	5	0	2	7
2025	472	45	1	2	48
2030	970	70	5.5	8	83
2035	1292	62	11	17	90
2040	1481	50	17	27	94
2045	1662	41	20	34	95
2050	1937	39	20	38	97

The PPA model was run for the scenarios described in Table 2, with results applicable to California, assuming LDV incentives available in 2022 remain effective until 2032. Market shares for various vehicle types were obtained and are discussed in Section 4. In this section, average market shares for LDVs are calculated and compared with the MA3T results. The PPA results for the base case, covering 2020–2040, are presented in Table 5. This base case assumes standard costs for batteries and fuel cells, as well as the infrastructure for battery charging and hydrogen refueling that would likely exist without significant intervention from California or federal authorities. The results show a steady increase in the market shares of battery-electric plug-in vehicles, projected to reach 82 % by 2040. In contrast, significant market development for FCVs is not anticipated until the late 2030s, aligning with the projections obtained from MA3T. Notably, the base case results for California suggest a faster growth in market share for plug-in vehicles than those projected by Oak Ridge Lab in Table 3 for the entire United States. This faster growth can be attributed to California's focused support for BEVs compared to most other states.

**Table 5:** LDV Base Scenario S1 with incentives to 2032.

Year	%BEV	%FC-HEV	%FCPHEV	%PHEV	%total ZEV
2020	7.6	0	0	2.0	9.6
2024	17.9	0	0	3.3	21.2
2028	38.4	0	0	5.1	43.5
2032	56.5	0	0	6.6	63.1
2036	73.6	0	0.4	7.4	81
2040	75.5	7.3	10.4	6.8	82.3

The MA3T results (Table 3) for California indicate a rapid growth of BEV market share similar to the PPA results in Table 5. The MA3T results consistently show a higher market share for PHEVs than in the PPA results. The reason for this difference should be investigated.

Table 6, Table 7, Table 8 present the results from the PPA model for Scenarios S2, S3, and S4. In these scenarios, the infrastructure for battery charging and hydrogen refueling is expanded to levels more comparable to those available for refueling gasoline ICEVs than in the base model (S1). Specifically, Scenario S2 focuses on improving battery charging infrastructure, Scenario S3 on enhancing hydrogen infrastructure, and Scenario S4 brings both infrastructures reasonably close to those for gasoline-fueled vehicles. The model results suggest that significant increases in market share for FCVs will not occur until the late 2030s, even with improved hydrogen infrastructure. This delay is primarily due to the fact that BEV technologies and markets are approximately 10 years ahead of those for hydrogen FCVs. In the long term, however, the model projects that the market shares for FCVs and BEVs will be comparable.

**Table 6:** LDV Scenario S2 with incentives to 2032.

Year	%BEV	%FC-HEV	%FCPHEV	%PHEV	%total ZEV
2020	7.6	0	0	2.1	9.7
2024	19.4	0	0	5.0	24.4
2028	41.6	0	0	5.3	46.9
2032	66.6	0	0	6.8	73.4
2036	84.6	0.7	1.8	7.4	94.5
2040	78.2	6.4	9.3	6.1	100

**Table 7:** LDV Scenario S3 with incentives to 2032.

Year	%BEV	%FC-HEV	%FCPHEV	%PHEV	%total ZEV
2020	7.6	0	0	2.1	9.7
2024	18.0	0	0	3.3	21.3
2028	36.6	0	0	5.3	41.9
2032	56.9	0.7	0.5	6.5	64.6
2036	69.6	10.1	6.7	7.0	93.3
2040	53.7	25.4	16.0	4.8	100

**Table 8:** LDV Scenario S4 with incentives to 2040.

Year	% BEV	%FC-HEV	%FCPHEV	%PHEV	% total ZEV
2020	8.1	0	0	2.1	10.3
2024	21.3	0	0	3.4	24.7
2028	44.1	0	0	5.3	49.4
2032	69	0.9	0.4	6.8	77.1
2036	75.5	11.8	6.3	6.3	100
2040	54.7	26.9	14.2	4.2	100

**Fig. 13:**
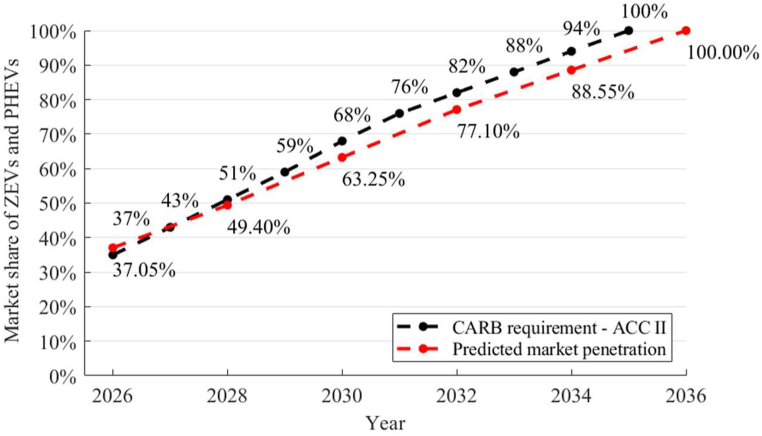
CARB ZEV requirements and the projection using the DDC model for LDVs.

The CARB proposed annual regulations for sales of ZEV LDVs are shown in Fig. 13 [6]. It can be observed that our PPA projections for scenario S4 (Low costs of energy storage devices, enhanced charging/refueling infrastructure, and incentives to 2040) closely align with the CARB proposed regulations – Advanced Clean Cars (ACC II).

## 5 Conclusions

The research discussed herein analyzes 14 decision factors influencing the purchases of cars, SUVs, and light-duty pickup trucks, particularly as they relate to ZEVs (BEVs, PHEVs, FCHEVs, FCPHEVs). We developed a DDC-based vehicle choice method for PPA to project ZEV market shares as vehicle and infrastructure technologies evolve from 2020 to 2040. Market share outcomes are presented for various scenarios based on different vehicle and infrastructure development strategies. The PPA findings suggest that BEVs of various sizes and classes are likely to achieve relatively high market shares by 2030 and have great potential to dominate the market up to 2040. For early adopters, model and brand attractiveness, as well as technological appeal, play key roles in breaking the ice, although they capture a relatively small market share initially. In the early years, incentives cannot generate significant market penetration of ZEVs, particularly for FCVs, due to the inconvenience of hydrogen refueling for consumers. Substantial incentives would have much greater impact in later years (after 2030) than in the early years, especially after the establishment of well-designed charging or hydrogen refueling infrastructure. Significant market penetration for hydrogen FCVs is not expected until the mid-2030s. For later adopters, lower vehicle costs, public fast charging, due to technological improvements, along with a sufficient range of models and brands to choose from, play important roles. Generally, the PPA outcomes suggest that concerted efforts to establish battery charging and hydrogen refueling infrastructure, alongside industry efforts to launch new ZEV models and government incentives, are crucial for meeting the CARB ZEV mandates for LDVs. Any of these measures is lost; it may affect the achievement of the target (100 % ZEV sales by 2035). Assumptions about infrastructure development and state policies are tailored to California, while those regarding ZEV development and costs have a global perspective. While the PPA method can be applied globally, the inputs for each decision factor must be carefully adjusted to reflect the specific conditions (e.g., vehicle technology, policy, infrastructure, etc.) of the country/region being studied.

## Data availability statement

Most of the data supporting the findings of this study are presented in the manuscript, including the Appendices. Other data are available from the corresponding author upon reasonable request.

## CRediT authorship contribution statement

**Andrew F. Burke:** Writing – review & editing, Writing – original draft, Project administration, Methodology, Investigation, Formal analysis, Conceptualization. **Jingyuan Zhao:** Writing – review & editing, Writing – original draft, Visualization, Software, Methodology, Investigation, Formal analysis. **Marshall R. Miller:** Writing – review & editing, Resources, Formal analysis. **Lewis M. Fulton:** Writing – review & editing, Supervision, Resources, Project administration.

## Declaration of competing interest

The authors declare that they have no known competing financial interests or personal relationships that could have appeared to influence the work reported in this paper.

## Figures and Tables

**Table A1:** Comparison of ORNL and UCD cost inputs for mid-size cars for 2025–2040 [12].

Powertrain technology	Source	2025	2030	2040	Markup
Conventional	ORNL	$26,068	$26,320	$26,403	1.6
	UCD	$26,500	$26,500	$26,500	
EV-200 miles	ORNL	$37,169	$32,443	$30,181	1.7–1.64
	UCD	$28,647	$25,046	$23,250	
EV-300 miles	ORNL	$44,248	$36,934	$33,214	1.7–1.68
	UCD	$31,700	$26,800	$24,300	
FC-400 miles	ORNL	$34,860	$33,800	$31,660	1.42–1.53
	UCD	$53,700	$42,500	$36,200	
PHEV-40 miles	ORNL	$39,238	$33,898	$33,214	1.6
	UCD	$32,300	$30,000	$28,500	

**Table A2a:** Summary of ZEV cost projections from UCD, ANL [70], and ICCT [71] for mid-size passenger car.

Mid-size passenger car (k$)	EV - 300 mi.			PHEV - 40 mi.		
Year	UCD	ANL	ICCT	UCD	ANL	ICCT
2022	34	47	39	34	42	39
2025	32	42	35	32	40	36
2030	27	35	28	30	34	34
2035	25	33	26	29	33	34

**Table A2b:** Summary of ZEV cost projections from UCD, ANL [70], and ICCT [71] for small SUV.

Crossover LDV/small SUV (k$)	EV - 300 mi.			PHEV - 40 mi.		
Year	UCD	ANL	ICCT	UCD	ANL	ICCT
2022	38	47	42	38	43	38
2025	35	42	36	35	39	34
2030	29	35	31	29	34	32
2035	27	34	28	27	33	31

**Table A2c:** Summary of ZEV cost projections from UCD, ANL [70], and ICCT [71] mid-SUV.

Sport Utility Vehicle/mid-SUV (k$)	EV - 300 mi.			PHEV - 40 mi.		
Year	UCD	ANL	ICCT	UCD	ANL	ICCT
2022	47	58	60	44	45	58
2025	43	49	52	43	42	56
2030	37	41	41	41	36	54
2035	35	40	40	40	35	52

**Table A2d:** Summary of ZEV cost projections from UCD, ANL [70], and ICCT [71] for LD pickup truck.

LD pickup truck (k$)	EV - 300 mi.			PHEV - 40 mi.		
Year	UCD	ANL	ICCT	UCD	ANL	ICCT
2022	49	68	58	45	50	54
2025	45	58	52	44	46	52
2030	38	47	44	40	39	50
2035	36	46	42	39	37	48

**Table A3a:** ZEV costs from ORNL/ANL for various types of vehicles [70].

Year	Car				CSUV			
	ICEV*	EV300	FC	PHEV40	Conv.	EV300	FC	PHEV40
2025	$26,080	$41,600	40,000	39,200	25,920	46,080	40,320	40,160
2030	$26,400	$34,720	35,680	33,920	26,240	38,720	36,480	34,080
2035	$26,560	$33,600	33,600	33,120	25,920	37,440	34,560	33,280
2040	$26,400	$33,120	33,120	32,320	25,760	37,120	33,920	33,280
2045	$26,240	$32,640	32,640	31,520	25,600	36,160	33,440	33,120

*veh cost = 1.6*ANL manuf. cost.

**Table A3b:** ZEV costs from ORNL/ANL for Light-duty pickup and midsize SUV [70].

Year	LD pickup				TSUV			
	ICEV	EV300	FC	PHEV40	ICEV	EV300	FC	PHEV40
2025	$29,920	$57,760	45,600	46,240	27,360	48,960	41,760	41,920
2030	$29,920	$47,200	40,960	38,720	27,520	41,120	38,080	35,840
2035	$30,080	$45,760	38,560	37,440	27,680	40,800	36,600	34,880
2040	$29,760	$45,280	37,920	37,280	27,360	40,480	35,360	34,880
2045	$29,280	$44,160	37,440	36,960	27,200	39,520	34,880	34,560

*veh cost = 1.6*ANL manuf. cost.

**Table A4a:** ZEV costs from UC Davis for midsize car and small SUV.

Year	Midsize car				Small SUV			
	ICEV	EV300	FC	PHEV40	ICEV	EV300	FC	PHEV40
2025	$26,500	$31,700	$40,300	$32,300	$28,000	$35,000	$41,700	$33,800
2030	$26,500	$26,800	$32,100	$30,000	$28,000	$29,200	$33,000	$31,500
2035	$26,500	$25,400	$28,400	$29,500	$28,000	$27,400	$29,600	$30,700
2040	$26,500	$24,300	$27,000	$28,500	$28,000	$26,000	$28,700	$30,400

**Table A4b:** ZEV costs from UC Davis for light-duty pickup and midsize SUV.

Year	LD Pickup				Midsize SUV			
	ICEV	EV300	FC	PHEV40	ICEV	EV300	FC	PHEV40
2025	$36,000	$44,600	$53,700	$43,500	$37,000	$42,900	$52,500	$42,800
2030	$36,000	$37,600	$42,500	$40,400	$37,000	$36,700	$42,900	$40,900
2035	$36,000	$35,100	$37,500	$39,000	$37,000	$34,600	$38,100	$39,600
2040	$36,000	$33,200	$36,200	$38,500	$37,000	$33,100	$36,600	$38,900

**Table A5:** Costs of BEVs on the market in 2022 from the official websites of the EV companies.

Car Model	Driving Range (EPA est.)	Type	MSRP Price (USD)
Tesla Model 3	272 - 333 miles	EV	$40,240 - $47,240
Chevrolet Bolt EUV	247 miles	EV	$28,795 - $33,790
Hyundai Kona EV	258 miles	EV	$33,550 - $41,550
Toyota Mirai	357–402 miles	FCHEV	$49,500 - $66,000
Honda Clarity	366 miles	FCHEV	Starting at $59,485
Hyundai Nexo	380 miles	FCHEV	Starting at $62,000
Chevrolet Volt	38 - 53 miles (all electric range)	PHEV	Starting at $34,395
Toyota Prius Prime	39 - 44 miles (all electric range)	PHEV	$32,350 - $39,170
Ford Escape PHEV	37 miles (all electric range)	PHEV	$27,500 - $32,340

**Table B1:** Battery pack costs for LDV in 2020–2040 (costs to OEMs).

Battery cost ($/kWh)	2020	2025	2030	2035	2040
- Hi cost case	180	140	110	90	70
- Base cost case	160	125	85	75	60
- Low cost case	140	100	75	60	50
Battery integration factor	1.2	1.15	1.11	1.05	1.05

**Table B2:** Fuel cell costs for LDVs in 2020–2040.

Fuel cell cost ($/kW)	2020	2025	2030	2035	2040
High cost case	300	135	85	60	50
Base case	225	100	70	50	45
Low cost case	175	60	50	45	40
Fuel cell system integration factor	1.2	1.15	1.1	1.1	1.05

**Table B3:** Costs for hydrogen storage on the vehicle in 2020–2040.

Year	$/kgH2
2020	1400
2025	800
2030	400
2035	350
2040	300

**Table B4:** Maintenance cost inputs for LDVs.

LD Vehicle Types	EV $/mile	FCV $/mile	ICEV $/mi
Mid-size car	0.031	0.05	0.062
Small SUV	0.031	0.05	0.062
Mid-size SUV	0.04	0.06	0.07
LD pickup	0.04	0.06	0.07

**Table B5:** Hydrogen costs ($/kg) for fuel cell vehicles produced from electrolysis.

Hydrogen cost	2020	2025	2030	2035	2040
High cost	17	12	9	7	6
Base (average)	12	8.5	7	6	5
Lower cost	10	7	6	5	4

**Fig. B1:**
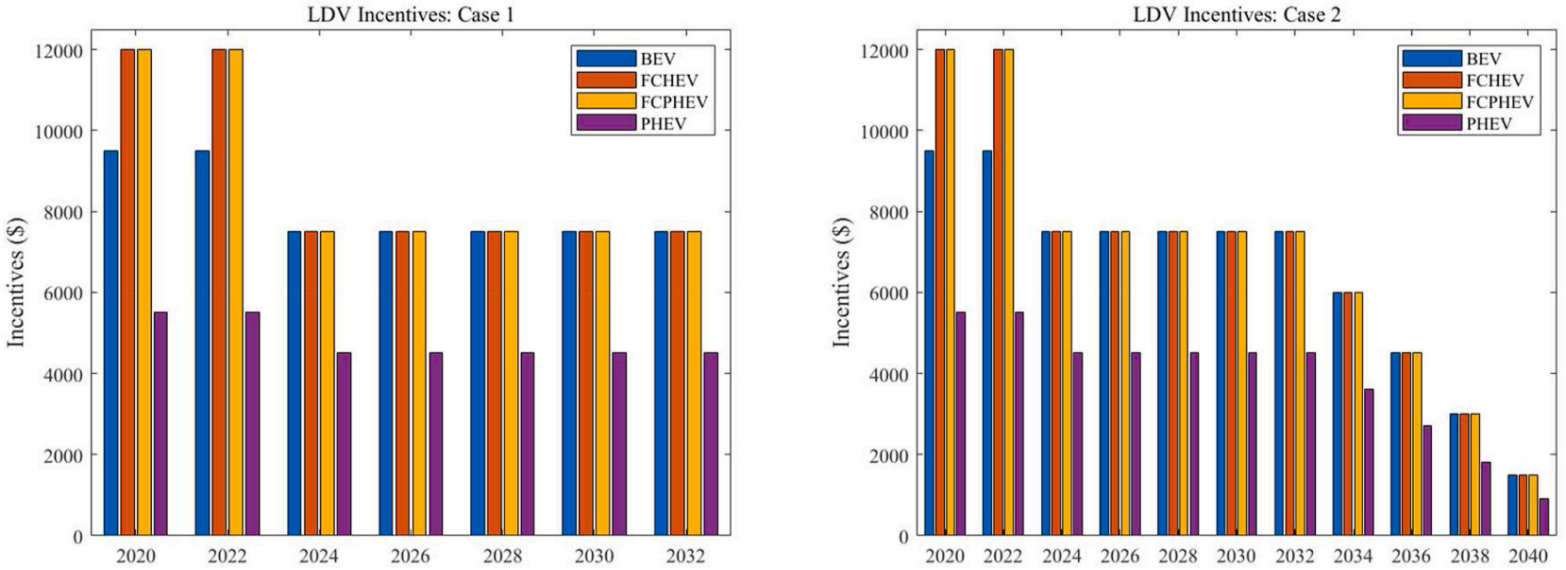
The two incentive strategies for LDVs are used in this study.

## Appendix A Definition and calculation of decision factors

### General comments

The purchase probabilities are analyzed in terms of sixteen decision factors. The influence of each decision factor is described in terms of a base value (bv), an input value (inpv), and an importance value (a). These quantities are used to calculate the P_r_ for each decision factor

(1) Pr = e^a(1-1/x)^

x = inpv/bv is the measure of the status of the vehicle being analyzed compared to the comparable ICE vehicle. The ratio is selected such that x < 1 for the early years and might increase to >1 in later years as inpv changes. x and P_r_ are calculated from the input values of bv, inpv, and “a” by the spreadsheet program for each decision factor as discussed in the following sections.
1. Vehicle cost

The purchase cost of each vehicle included in this analysis has been calculated in Ref. [40].

(2) Vehicle_cost = Glider cost + cost of the powertrain and energy storage components

The glider cost was determined by subtracting the ICE cost from the cost of the ICE vehicle. The powertrain and energy storage and conversion components were sized to achieve the desired acceleration and range for each vehicle type and ZEV option-BEV, FCV, and PHEV. The cost of each component was calculated based on its size and unit cost. For example, battery cost = (kWh)x($/kWh), fuel cell cost = (kW)x($/kW), electric motor cost = (kW)x($/kW), hydrogen storage unit cost= (kgH2)x($/kgH2). The unit costs of the electric drive, batteries, and fuel cells were reduced systematically from 2020 to 2040 to reflect maturing of the technologies. The cost of integrating the various components into the driveline was done by multiplying the cost of the component by a factor (1+Δ). Δ was decreased as the technologies matured. The details of all these vehicle cost calculations are given in Ref. [40] and in Appendix B for the costs of batteries and fuel cells.

For the vehicle cost decision factor,

(3) x = Cost_ICE_/C_ZEV_

The base vehicle cost bv is in all cases the cost of the comparable ICE vehicle. Selecting an appropriate” a” value for the vehicle cost factor is important especially in the early years before 2030 when the cost of ZEVs can be relatively high. “a” between 1 and 10 seems like a reasonable choice.

### Costs of ZEV LDVs

The UCD projected costs of BEVs and FCVs for 2020–2040 [39] are compared with the corresponding costs published by Oak Ridge National Laboratory (ORNL) [12] and DOE Argonne National Laboratory (ANL) [70] and in Table 1, Table 2, Table 3 for LDVs. Vehicle costs are shown for various powertrain types, including BEV-300, FC-HEV, and PHEV-40. In the case of BEV and PHEV, the projected UCD costs are somewhat lower than those of both ANL and ORL for the next two decades (2020–2040). However, for FC-HEV, our costs are higher than both ANL and ORL values in the 2020s, but they converge and become similar as we approach 2040. The UCD projections exhibit a larger, steady decrease in ZEV costs up to 2030 and beyond. The ANL approach to estimating future costs involves calculating the manufacturing cost of the ZEV and then applying an assumed markup factor to determine the purchase price of the vehicle. On the other hand, in the UCD analysis of ZEV costs (Table A4), it was assumed that all component costs (batteries, fuel cell, and electric driveline) were retail costs with no markup applied. As shown in Table 1, Table 2, Table 3, Table 4, these different approaches result in similar but somewhat different cost values. Table A5 gives the costs of selected BEV and FCV models available on the market in 2022 indicating that the UCD cost projections may prove to be reasonable for future years.

2. All-electric or hydrogen range (mi)

The range of a BEV is determined primarily by the size (kWh) of the battery in the vehicle. Range was a key factor in the cost of EVs when batteries cost $200–300/kwh and higher, but now that battery costs are much lower the effect of range on EV cost is of less concern. Electric LDV vehicles with ranges of 300–400 miles are on the market. For this study, 300 miles has been selected as the base range for the BEVs. This is significantly less than that of ICEVs and the battery charging time is relatively long even at fast chargers, but EV sales indicate that 300 miles seems to be acceptable to many buyers. Our EV cost studies indicate that 300 miles permits cost equity with ICEVs by 2030.

The range of a FCV depends primarily on the kgH2 stored onboard the vehicle. Hydrogen storage limits are set by the volume available for the H2 storage tanks. Hydrogen storage is expensive, but not nearly as expensive as for electricity in batteries. Fuel cell passenger cars on the market currently have ranges of 300–400 miles. The hydrogen refilling time for FCVs is comparable to that of ICE vehicles. 400 miles has been selected for the base range of the light-duty FCVs in this study. That range should result in near equity in FCV costs with ICEVs in 2030 and beyond as fuel cell technology matures.

For the range decision factor, x = range ZEV/range ICE, where Range ICE = 500 miles, Range BEV = 300 miles, range FC = 400 miles. An “a” value of 1–2 seems appropriate as the ZEV ranges are not an important issue for class 3–7 at the present time.

3. Number of ZEV models available to purchase

In the early years, the small number of ZEV models available to purchase is an important issue. At the present time, there seems no doubt that auto industry will rapidly provide many new models of BEVs. However, there are only a few FCV models on the market and the present prospects for many more in the near future are not promising. Setting the input values for the model decision factor is difficult. The approach taken in this study was to set an arbitrary number such as 25 or 50 for the base number for ICEVs and then to think in terms of the fraction of the base value for the input value for the ZEV options. In the case of BEVs, the model number is getting relatively large, approaching the base, while for FCVs the model number is a small fraction of the base number. For the model number decision factor, x = number of ZEV models/base number of modeled needed.

4. Inconvenience to charge or refuel ZEVs in the city compared to ICEV

This decision factor is concerned with refueling ZEVs for short trips in the city and its influence on the purchase decision. Refueling with electricity is basically different than with hydrogen so battery-electric and hydrogen FCVs will be considered separately.

### Chargers for BEVs

BEVs used by people living in single-family homes can be charged conveniently over-night at home. Hence, a significant fraction (50–75 %) of the early EV users have home charging. People without home charging will have to depend on public charging facilities. For these people, they will need chargers relatively close to their homes for convenient charging of their BEV. The question of proper city infrastructure for BEVs deals with how many public fast chargers are needed so those without home charging can conveniently charge their vehicles. The question is further complicated because it is expected that those with home charging will occasionally need to use public chargers.

All the chargers are 300 kW with 2-plugs capable of charging the vehicles in 20 min or less. The goal of the battery charging infrastructure is to make battery charging as convenient as possible compared to refilling a vehicle with gasoline. There are about 8000 gasoline stations with an average of 8 pumps in California to refill about 25 million vehicles. Hence, it is assumed that the number of chargers needed is to match gasoline refilling convenience. The ‘8.3 factor” is to account for the time it takes to charge the battery and the more times the battery is charged compared to refueling the ICE vehicle. The number of chargers needed for 1 and 5 million BEVs are shown below.

(4) Number chargers = 8 × 8 x 8.3 × .5 10^3^ /25 million BEVs

(5) 1 million BEVs No. of chargers = 8 × 8 × 8.3 x .5 × 1/25 × 10^3^ = 10624

(6) 5 million BEVs No. of chargers = 8 × 8 × 8.3 x .5 × 5/25 × 10^3^ = 53120

We need to account for home charging in the formula for “x”.

(7) x = x_home_ + number chgr/base chgr, x_home_ = 0.4

It also seems reasonable to pre-build about 10 % of the total chargers needed by 2022. The change in number of chargers available in 2020–2040 depends on the strategy in building the charger infrastructure. The base value depends on the number of BEVs expected to be on the road in 2040.

### Hydrogen stations/dispensers for FCVs

Hydrogen stations are needed to refuel all the FC vehicles on the road. The typical mid-size FCV will use about 168 kgH2/yr. so the H2 demand at the stations will be 168 million kgH2/yr./million vehicles. If the average H2 station dispenses 1000 kg/day with a utilization factor of 0.5, the number of H2 stations needed for 1 million FC vehicles will be the following.

(8) Number of 1000 kgH2/da sta. = 168 × 10^6^ /(365 × 1000 x .5) = 920

We could have fewer stations if they are larger, or we assume a higher utilization factor say 0.7. The California Partnership is projecting 1000H2 stations and 1 million FC vehicles for 2030.

Assuming each refilling is 5 kg, that is 34 refilling per year. The base values for H2 stations is then

(9) 1 million FCVs 920 sta. 2–3 dispensers

(10) 5 million FCVs 4600 sta. 2–3 dispensers

For the H2 stations,

(11) x = number of H2 stations/ base value for stations

We need to pre-build 10 % of the total stations needed in 2030.

5. Inconvenience to charge or refuel ZEV on the highway compared to ICEV

All BEV owners will need public chargers for long trip travel beyond the range of their vehicle. This will require the availability of fast chargers at which they can get a significant charge (at least 65 % of battery kWh capacity in 20 min or less). The FCV owners will need hydrogen stations located along highways much like present gasoline stations as the hydrogen refueling times are comparable. The charger and H2 station projections made in the previous section for decision factor 4 include satisfying refuel needs for long trips. The “x” values will be determined using the same formula as for short trips.

6. Battery charging or hydrogen refueling time (minutes) compared to the ICEV

As noted previously, the charging times of BEVs at public chargers will be much longer than for refueling hydrogen FCVs at public H2 stations. This difference will be included in the calculation of “x” and P_r_ for the BEV and FCVs. At the present time (2022), the minimum time for fast charging batteries is 30–40 min with the charge (kWh) added being 65 % or less of the battery kWh capacity. In the future with improvements in battery technology, it seems likely that the minimum fast charging time will be reduced to 15 min or less. Hence the base value for fast charging BEVs is set at 15 min and the input value will be decreased from 40 to 15 min from 2020 to 2040. Hence

(12) x = input min charging time/15 for BEVs

In the case of FCVs, it is assumed the refill time with H2 will be essentially the same as with gasoline in ICEV2. Hence, x = 1 for all FCV using hydrogen.

7. Availability of a second market for ZEVs compared to ICEVs

The availability of a second market for ZEVs is critical to the development of the mass market for them. There seems little doubt that a second market similar to that available for ICEVs will develop as their market fraction and number of ZEVs on the road increases in the future. It will take time for the second market to develop. This is included in the probability calculations by gradually increasing the x value of decision factor 7 over the time period 2020–2040.

8. Maintenance cost ($/mi) of ZEVs compared to ICEVs

The maintenance cost was included in the economic analysis of the ZEVs in Ref. [40]. The maintenance values are based on and given as $/mi. For each type of vehicle, the maintenance value is calculated by dividing the annual maintenance cost by the annual mileage. The base value is the maintenance cost of the ICE vehicle. The x values of maintenance of the ZEVs are assumed to decrease in future years as the technologies mature. Hence,

(13) x= ($/mi)_ZEV_ /($/mi)_ICEV_

for both BEVs and FCVs.

9. Energy operating cost ($/mi) of ZEVs compared to ICEVs

The energy operating cost was calculated in the economic analysis of the ZEVs in Ref. [40]. The energy cost values ($/mi) are based on the energy used per mi (kWh/mi or kgH2/mi) and the cost of energy ($/kWh or $/kgH2). The energy cost values decrease in future years for all the vehicles as the technologies mature (energy/mi decreases) and the price of energy, especially hydrogen, is reduced.

The base value is the energy cost ($/mi) of the ICE vehicle The value of x for energy use is given by

(14) x= ($/mi)_ICE_ /($/mi)_ZEV_

for both BEVs and FCVs. x in the early years is less than 1, but later years x can be significantly greater than 1. In this case, the value P_r_ is also greater than 1 and decision factor 9 can have a big effect on the average purchase probability of the vehicle.

10. Environmental concern of ZEV buyers compared to ICEV buyers

The regulated and GHG emissions of ZEVs are very low, essentially zero. To some potential vehicle purchasers, the reduced environmental/climate change impact of ZEVs is very important. In that case, the value of x for ZEVs and the resultant probability would be greater than 1. It is assumed that x > 1 for all ZEVs and years and that it increases from 2020 to 2040 as the general public gains a greater understanding of climate change and the need to reduce GHG emissions. The base value is 1 for all vehicles. x < 1 for any vehicle with an ICE even PHEVs. x is defined as

(15) x= (envir. concern)_ZEV_ /(envir. concern)_ICE__base_

Setting values for this decision factor is subjective, but important nevertheless.

11. Safety concerns for ZEVs compared to ICEVs

There are safety concerns about both batteries [72,73] and hydrogen [74,75]. In the case of batteries, there are concerns about battery fires and explosions resulting from thermal runway of one or more cells in the battery pack. Such occurrences have been relatively rare, but they can be a concern for some vehicle buyers. In the case of FCVs, the use of hydrogen as the fuel brings to mind the Hindenburg airship accident in 1937. There is no doubt that H2 must be handled with care to prevent fires and explosions, but over the years, hydrogen has been handled safely in many industrial applications. These applications include considerable safe experience with FCVs in both the past and present. Gasoline is a flammable fuel and fires in **gasoline** fueled vehicles occur with relative rarity. Safety is a critical factor in the development of both battery and fuel cell systems. It seems clear we can assume both the ZEVs are relatively safe now and will become safer in the future.

12. Drivability of ZEVs compared to ICEVs

One of the key advantages of ZEVs compared to engine/transmission powered ICEVs is their excellent performance and drivability. ZEVs have fast, smooth acceleration and very quiet operation. The electric driveline has very fast response and high efficiency. After a demonstration drive in a BEV, people express how impressed they are with its drivability. They always rate the drivability of the BEV much superior to the ICEV they customarily drive. The “x” value for this decision factor is defined as

(16) x_12_ = (drivability)_ZEV_ /(drivability)_ICEV_

“x” will be > 1 in all cases for ZEVs. In later years, “x” for the ICEV will be < 1 as people's drivability expectations become closer to that of BEVs.

13 Reliability/durability of ZEVs compared to ICEVs

Purchasers of ZEVs will be concerned about the reliability/durability of their vehicles using new technologies. They will want their ZEVs to have reliability/durability comparable to the ICEVs. That may not be the case in the early years, but as the technologies mature, it will be true in later years. A primary issue concern for ZEVs is the aging and reliability of energy storage and powertrain systems, such as batteries [[76], [77], [78], [79]] and fuel cells [80,81].

The “x” values used in the probability tables for the ZEVs will reflect their improved durability in future years. x will be < 1 in the early years and should approach 1 in the later years. This certainly the case for first car buyers, but the long-term durability for both batteries and fuel cells is still uncertain. The x for durability is defined as

(17) x_13_ = (Veh dur)_Zev_ / (veh dur)_ICEV_

14 Excitement with new technology

In the early years, excitement with the new technology of battery-electric drive [82] and fuel cells [83] can be an important consideration in the decision to purchase a ZEV. This factor will not be important to all potential vehicle buyers, but to some it could be very important. ICEVs are an old technology, and the public seems ready for a new one. The “x” value is defined as

(18) x_14_ =(excitement)_ZEV_ /(excitement)_ICEV_

“x” is > 1 in all cases for ZEVs. “x” for ICEVs will be < 1 in later years.

## Appendix B Key inputs assumed in the ZEV cost calculations

Some of the decision factors (especially the financial factors) are numerical in character and are based on economic evaluations of ZEVs [39]. The results obtained from these evaluations are highly dependent on the inputs used in the economic analyzes of the vehicles. In this appendix, some of the most critical inputs are presented. These inputs include battery and fuel cell costs, hydrogen costs, and maintenance costs.

1. Battery and fuel cell costs

The cost of the BEVs is strongly affected by the cost ($/kWh) of the batteries. The costs ($/kWh) of the batteries assumed in the vehicle cost calculations for LDVs are given in Table B1. Three cost levels are given for each year-high, base, and low. The battery costs are assumed to decrease between 2020 and 2040 as the battery technology matures. The battery in each vehicle analyzed was sized based on its kWh/mi and design range. Hence the effect of battery cost on vehicle cost was greater for long range vehicles. An additional battery cost was included to account for integrating the battery into the vehicle's powertrain.

The cost of the FCVs is strongly affected by the cost ($/kW) of the fuel cells and hydrogen storage ($/kgH2) unit. The costs ($/kW) of the fuel cell system assumed in the vehicle cost calculations for LDVs are given in Table B2. Three cost levels are given for each year-high, base, and low. The fuel cell costs are assumed to decrease between 2020 and 2040 as the fuel cell technology matures. The cost of FCV can be strongly affected by the size kW of the fuel cell in high performance vehicles. The assumed costs ($/kgH2) of the energy storage unit are given in Table B3. The energy storage unit is sized (kgH2) by the kgH2/mi of the vehicle and its design range. The vehicle cost increases with the range of the FCV, but the effect is smaller than for batteries in BEVs. As for batteries, an additional cost was included to account for integrating the fuel cell system components into the vehicle's powertrain.

2. Vehicle maintenance

Calculation of the TCO values for the vehicles required inputs for their maintenance ($/mi). Information on maintenance especially for the ZEVs is difficult to find. The maintenance values used in this study are shown in Table B4.

3. Cost of hydrogen

The energy cost ($/mi) to operate FCVs depends on the cost of hydrogen ($/kgH2). The cost of H2 is presently very high, but it is expected to become much lower in the future. Projected hydrogen cost is given in Table B5. In the present study the base value of H2 was to calculate the TCO of the FCVs.

4. Financial incentives

The financial incentives from 2020 to 2032 will be based on the current policy, which includes CVTC [66] and CVRP [67]. The incentives for Plan 1 and 2 are shown in Figure B1. For Plan 2, from 2032 until 2040, the incentives will gradually decrease at a constant rate each year.
